# Olfactory epithelium organoid models identify Ddit3 as a potential therapeutic target against inflammation-related olfactory sensory neuronal loss and functional deficit

**DOI:** 10.7150/ijbs.129192

**Published:** 2026-03-30

**Authors:** Jinxia Liu, Jiaming Qi, Nan Jiang, Yuzhen Wang, Weihao Li, Shiyi Tian, Liujing Zhuang, Yunfeng Zhang, Yongliang Liu, Yiqun Yu

**Affiliations:** 1 ENT Institute and Department of Otorhinolaryngology, Eye & ENT Hospital, Fudan University, Shanghai, China.; 2 Olfactory Disorder Diagnosis and Treatment Center, Eye & ENT Hospital, Fudan University, Shanghai, China.; 3 Biosensor National Special Laboratory, Key Laboratory for Biomedical Engineering of Education Ministry, Department of Biomedical Engineering, Zhejiang University, Hangzhou, 310027, China.; 4 School of Food Science and Biotechnology, Zhejiang Gongshang University, Zhejiang 310018, China.; 5 Key Laboratory of Animal Biodiversity Conservation and Integrated Pest Management, Chinese Academy of Sciences, Beijing 100101, China.; 6 University of Chinese Academy of Sciences, Beijing 100101, China.; 7 Department of Otolaryngology, Zibo Central Hospital, Zibo, Shandong, China.

**Keywords:** olfactory epithelium, organoid, olfactory sensory neuron, inflammation, Ddit3, odor response

## Abstract

**Background:**

Inflammatory activation is a major cause to nasal diseases, such as chronic rhinosinusitis and allergic rhinitis. However, in vitro research model to mimic the process of olfactory inflammation and to screen new therapeutic target is still lacking.

**Methods:**

We established three inflammatory models based on olfactory epithelium (OE) organoids, using lipopolysaccharide (LPS), TNFα treatment and doxycycline induction. The efficacy of these models was evaluated by immunostaining, RNA sequencing, qPCR, and functional assays.

**Results:**

These inflammatory organoid models mimicked impairment in cell proliferation and neuronal genesis, and showed upregulation of inflammation-related signaling pathway and downregulation of cell cycle-related pathway. We identified that DNA damage inducible transcript 3 (Ddit3) was upregulated in all inflammatory organoid models. Ddit3 downregulation counteracted apoptosis, alleviated cell proliferation and neuronal differentiation, and recovered the functional response to odor stimulation in all three inflammatory organoid models. Ddit3 deficiency counteracted effect of LPS instillation by promoting cell proliferation, recovering neurogenesis, attenuating inflammation, and improving electrophysiological response to odor mixes in the OE. Single-cell RNA sequencing analysis showed that Ddit3 upregulation in mature olfactory sensory neurons of inducible inflammation model and patients with aging-related olfactory dysfunction correlated with endoplasmic reticulum stress and neuron apoptotic process.

**Conclusions:**

We established olfactory inflammation organoid models, and made use of these models to identify Ddit3 as a potential therapeutic target against inflammation-related olfactory neuronal loss and functional deficit.

## Introduction

The sense of smell is crucial for perception of external dangers and maintaining physical health^1^. The olfactory epithelium (OE) is a key olfactory organ with self-renewing and regenerative capacity throughout the lifespan^2^, predominantly constituted by basal cells, olfactory sensory neurons (OSNs), and sustentacular cells. Changes in the OE such as declined proliferative capacity in basal cells^3^ and chronic inflammation^4^ cause epithelial reorganization and olfactory functional impairment. Olfactory dysfunction (OD) is one of the common nasal diseases, and inflammatory process is a major etiological factor leading to the occurrence of OD^5^. Mouse models with upper respiratory inflammation and OD showed significant reductions in the number of mature and immature OSNs^6^. OD is a common and clinically significant issue in patients with upper respiratory inflammatory diseases, such as chronic rhinosinusitis (CRS) and allergic rhinitis (AR). However, there is no efficient therapy against olfactory inflammation to recover the neurogenesis and functional capacity.

Currently, olfactory inflammation model is still lacking in the related research field. A commonly used model is lipopolysaccharide (LPS)-initiated chronic olfactory inflammation to mimic the pathological condition such as CRS^7^. In a mouse genetic model, the chronic inflammation was controlled by doxycycline (DOX) induction, leading to infiltration of inflammatory cells into the OE and functional impairment^4^. These features mimic essential aspects of CRS-associated olfactory loss. However, there is still lack of a convenient and effective *in vitro* model to capture the process of olfactory inflammation. In past few years, organoid technology is widely applied in studying developmental processes, disease modeling, infection dynamics, and cancer treatment responses^8^. Three dimensional cultured organoids were established by using progenitor cells isolated from olfactory epithelial tissue^9^-^11^. This OE organoid model was used to reveal the roles of critical genes in OE homeostasis^12^, ^13^, regeneration^14^, ^15^, and aging process^16^. These reports fully provide evidence that OE organoid is an ideal model to mimic the real tissue and elucidate the gene function. However, there is still no report showing establishment of an olfactory inflammation organoid model.

Inflammation plays complex roles in the OE. Transient increase in cytokine levels, such as tumor necrosis factor (TNFα) and interleukin (IL)-1β by acute inflammation was required for the regenerative process^17^. However, chronic activation made these stem cells cease their regenerative activity and instead signal macrophages to sustain immune defense in the OE^18^. In inflammatory nasal diseases such as CRS, understanding how the transcriptional landscape changes may identify therapeutic targets against inflammation and recover the olfactory function. In the present study, we established three inflammatory OE organoids based on LPS instillation, TNFα treatment, and DOX induction. Inflammatory OE organoid models showed the impaired cell proliferation and neuronal differentiation. Bulk RNA sequencing (RNA-seq) analysis revealed upregulation of inflammation-related signaling pathways and downregulation of cell cycle-related pathway, and identified upregulation of Ddit3 in all three organoid models. Knockdown of Ddit3 in inflammatory OE organoid models alleviated apoptosis, rescued cell proliferation, OSN generation, and functional response to odor stimulation. Furthermore, Ddit3 deficiency recovered cell proliferation, neurogenesis and electrophysiological response to odor mixes, and weakened inflammation in the OE of LPS-instilled mouse model. Single-cell RNA sequencing (scRNA-seq) analysis revealed that Ddit3 upregulation in mature OSNs of inducible olfactory inflammation model correlated with endoplasmic reticulum (ER) stress and neuron apoptotic process, and DDIT3 in mature OSNs of patients with aging-related olfactory function showed the same regulatory activity. Collectively, we successfully established olfactory inflammation models based on OE organoid platform, and revealed Ddit3 as a potential therapeutic target against olfactory inflammation and related cellular and functional deficits.

## Methods

### Animals

Wild type (WT), Ddit3^-/-^ (C57BL/6J background), and TRE-TNFα/Cyp2g1-rtTA mice were purchased from GemPharmatech Corp. (Nanjing, China). All animals were housed in specific pathogen-free grade experimental platform of the Animal Facility in Shanghai Medical Collage, Fudan University. To induce Chronic Rhinosinusitis (CRS) mouse model, WT and Ddit3^-/-^ mice (3-months aged) were instilled with lipopolysaccharide (LPS, 1µg/10g weight) intranasally for 7 days, then the mice were used for OE dissection, organoid culture and immunostaining. Inducible olfactory inflammation mice model (TRE-TNFα/Cyp2g1-rtTA) was established via crossing pTRE(3G)-mTnf-P2A-luciference-PolyA and Cyp2g1-P2A-rtTA(3G) strain and identified by PCR, based on the Tet-on genetic system. Equal numbers of male and female mice were randomly assigned to control and doxycycline (DOX) groups, DOX were added in food (0.625g/kg) for 4 months, starting at 3 months of age. Then the mice were used for OE dissection, organoid culture, and immunostaining. The procedures of animal breeding and tissue harvesting were approved by the Institutional Animal Care and Use Committee in Eye & ENT Hospital, Fudan University (Permit number: IACUC-DWZX-2025-038).

### Organoid culture and chemical treatment

Olfactory epithelial tissues were isolated and digested with 0.25% trypsin-EDTA for 30 min at 37 °C to obtain singe cell suspension, and digestion was terminated via adding DMEM/F12 medium with 10% FBS. 5 x 10^4^ cells were embedded in Matrigel (Corning, #35420) drops and seeded in 24-well plate (40 µl/well). Matrigel drops were polymerized at 37 °C for 30 min, and then 500 µl growth medium was added gently along wall into wells. The growth medium was based on DMEM/F-12 medium (ThermoFisher Scientific, #10565018) supplemented with R-Spondin-1 (200 ng/ml; R&D, #4645-RS), Noggin (100 ng/ml; PeproTech, #250-38), Wnt3a (50 ng/ml; R&D Systems, #5036-WN-010), , 1% N2 (ThermoFisher Scientific, #17502048), 2% B27 (ThermoFisher Scientific, #17504044), and 1% penicillin/streptomycin (Gibco, #15140-122), human epidermal growth factor (50 ng/ml, ThermoFisher Scientific, #PHG0311), Y27632 (10 µM, MCE, #HY-10583), HEPES (10 mM, Gibco, #15630-080), and Primocin (100 µg/mL, Invivogen, #ant-pm-05, added at Passage 0 only). For organoid passaging, Matrigel drops were gently rinsed with pre-cold cell recovery solution (Corning, #354253). Organoids were harvested and enzymatically digested with Organoid Dissociation Reagent (Precedo, #PRS-ODR) at 37 °C for 6-8 min to generate a single-cell suspension, and then were embedded with Matrigel. Optimal passaging ranged from 1:4 to 1:8, depending on organoid confluence. After 7-10 days of expansion, growth medium was replaced by differentiation medium containing LY411575 (5 µM, Sigma-Aldrich, #SML0506), SB431542 (10 µM, MCE, #HY-10431), and retinoic acid (5 ng/ml, Sigma-Aldrich, #R2625). The medium was replaced every 3 days.

After culturing for 7-14 days, recombinant mouse TNFα protein (Peprotech, #410-MT) was added into differentiation medium at various concentration (1, 5, 10 ng/ml) for 24, 48, and 72 h respectively. For LPS treatment, 1 µg/mL LPS (Sigma, #L2654) was supplemented into differentiation medium and maintained for 7 days. Organoids derived from the OE of TRE-TNFα/Cyp2g1-rtTA mice (age of 4-6 weeks) were cultured for 7-14 days, and then DOX (10 µg/ml, MCE, #HY-N0565B) was added for 24 h. Organoids were then harvested for RNA sequencing, immunostaining and functional assays.

### Cryosection preparation and immunostaining

Following induction of surgical-plane anesthesia in mice, transcardial perfusion with PBS and 4% paraformaldehyde (PFA) was conducted. Dissected heads were fixed in 4% PFA overnight at 4 °C, decalcified using 0.5M EDTA (pH 7.4) at 4 °C for 4-6 days, dehydrated in 10%, 20%, and 30% sucrose at 4 °C, and then embedded with TissueTek optimal cutting temperature (O.C.T.) compound. Sections at 20 µm were prepared by a Cryostat (model CM1950, Leica). For cultured organoids, they were incubated with cell recovery solution for 30 min on ice, fixed with 4% PFA for 45 min on ice, and then dehydrated in 30% sucrose at 4°C overnight. Organoids were embedded in pre-warmed gelatin/sucrose solution, equilibrated at 37°C for 15 min, and solidified at -20 °C. The solidified organoids were wrapped with O.C.T. compound, and then cut into 20 µm sections.

For immunostaining, the slides were baked at 60 °C for 1 h to enhance adhesion, washed three times with PBS (5 min each), blocked non-specific binding by BSAT (5% bovine serum albumin in PBS with 0.3% Triton X-100) at room temperature for 1 h, and then incubated with primary antibody at 4 °C overnight. Primary antibodies used here included rabbit anti-OMP (#ab183947, Abcam, 1:200), mouse anti-Ki67 (#550609, BD Biosciences, 1:200), goat anti-ICAM1 (#AF796, R&D Systems, 1:200), goat anti-IL33 (#AF-3626, R&D Systems, 1:200), rabbit anti-F4/80 (#GB113373, Servicebio, 1:200), rabbit anti-CD45 (#ab10558, Abcam, 1:200), rabbit anti-Ddit3 (#15204-1-AP, Proteintech, 1:100), rabbit anti-Top2a (#ab52934, Abcam, 1:200). After washed with PBST (0.3% Triton X-100 in PBS), the slides were incubated with Alexa Fluor 488 Donkey anti-Rabbit (#Invitrogen, A21206), Alexa Fluor 594 Donkey anti-Mouse (#Invitrogen, A-21203), Alexa Fluor 594 Donkey anti-Goat (#Invitrogen, A11058), Alexa Fluor 488 Donkey anti-Mouse (#Invitrogen, A21202), Alexa Fluor 568 Donkey anti-rabbit (#Invitrogen, A10042), Alexa Fluor 647 Donkey anti-rabbit IgG (#Invitrogen, A31573), or Alexa Fluor 488 Donkey anti-Goat (#Invitrogen, A11055) for 1 h at room temperature. All the secondary antibodies were diluted at 1:300 in BSAT. After washed with PBST by three times, nuclei were stained by DAPI. Fluorescent images were captured using a confocal microscope (Model SP8, Leica) with LAS AF Lite software.

### RNA sequencing

OE organoids (3-5 wells per group) were incubated with cell recovery solution for 30 min on ice, and rinsed by DMEM/F12 medium with 10% FBS. Total RNA was extracted using Trizol reagent kit (Invitrogen, Carlsbad, CA, USA) according to the manufacturer's protocol. The resulting cDNA library was sequenced using Illumina Novaseq6000 by Gene Denovo Biotechnology Co. (Guangzhou, China). Differentially expressed gene analysis was performed by DESeq2. The genes/transcripts with the parameter of false discovery rate (FDR) or P value below 0.05 and absolute fold change ≥ 2 were considered as differentially expressed. KEGG database was utilized to perform pathway enrichment analysis. Volcano plots and hierarchical clustering heatmap analyses were visualized using the Gene Denovo website (https://cloud.omicsmart.com).

### RNA extraction and quantitative PCR

Total RNA was extracted using an RNA isolation Kit (Vazyme, RC113), and the concentration of total RNA was determined by examining OD260/OD280 using Nanodrop. 500ng of total RNA was reverse transcribed into cDNA using HiScript II Q Select RT SuperMix for qPCR Kits (Vazyme, R233). Real-time PCR was performed using ChamQ Blue Universal SYBR qPCR Master Mix (Vazyme, Q312) on a Biorad CFX Opus 96 Real-Time PCR System. The relative expression of the target genes in different groups was normalized to GAPDH, and was determined using 2^-ΔΔCt^. The experiments were performed on triplicate.

### Adeno-associated virus preparation and infection

Plasmids expressing three different short hairpin RNA (shRNA) targeting different regions of the Ddit3 sequence were prepared by Genecloudbio (Guangzhou, China). The downregulation efficiency was determined via qPCR in HEK293 cells expressing mouse Ddit3, and shRNA with highest knockdown efficiency (GGTCCTGTCCTCAGATGAAAT) was employed to construct AAV-shDdit3. AAVDJ-shNC-EGFP and AAVDJ-shDdit3-EGFP were generated by PackGene Biotechnology (Guangzhou, China). Organoids cultured in differentiation medium were infected with AAVDJ-shNC-EGFP or AAVDJ-shDdit3-EGFP (1.0×10^10^ Vector Genomes) at Day 8, and the medium was replaced in 48 h. Organoids were maintained for another 7-10 days post infection, and then subjected to subsequent experiments.

### TUNEL assay

TUNEL assay was performed to analyze apoptosis in organoids. Fixed cryosection was washed twice with PBS (10 min each), and then incubated using PBS with 0.3% Triton X-100 at room temperature for 5 min. Subsequently, the slides were incubated with prepared TUNEL reaction mixture (Beyotime, #C1086, China) for 1 hour at 37°C in the dark, and iamges were captured using a confocal microscope (Model SP8, Leica) with LAS AF Lite software.

### Calcium imaging

Cultured organoids were incubated in loading buffer (124 mM NaCl, 3 mM KCl, 1.3 mM MgSO₄, 2 mM CaCl₂, 26 mM NaHCO₃, 1.25 mM NaH₂PO₄, 15 mM glucose) containing 4 μM Fura-2 AM (Invitrogen) and 0.02% Pluronic F-127 (Invitrogen) at 4 °C for 30 min in darkness to facilitate dye loading. Calcium imaging was performed using a Leica TCS SP8 confocal microscope (Leica Microsystems, Wetzlar, Germany), and parameters were as following: excitation at 340/380 nm, emission at 510 nm, acquisition rate of 2 s/frame, and maintained buffer flow during stimulation. Odor mix (odor mix1 contained eugenol, geraniol, allyl phenylacetate, 1-octanol, benzyl acetate, (R)-(-)-carvone, 2-heptanone, and citral, while mix2 included octanoic acid, heptanoic acid, coumarin, linalool, octanal, β-citronellol, (S)-(+)-carvone, and trans-cinnamaldehyde) or single odorant including 2-heptanone, 1-octanol, citral, eugenol stimulation lasted 15 s followed by 10-min washout with perfusion buffer. Calcium flux was quantified as ΔF/F0 (where F0 represents baseline fluorescence intensity).

### Microelectrode array recordings and signal analysis

In vitro electrophysiological recordings of OE tissues were made using a microelectrode array (MEA) system (MEA2100, Multi Channel Systems). After incubation, the tissue was mounted onto a custom-designed planar MEA (60 electrodes, 200 μm spacing, 30 μm diameter). In order to improve the contact of tissue to the electrodes, the tissue was held in place with a slice anchor (ALA HSG-5BD, glass-coated steel ring with polymidde-coated silica fibers, Multi Channel Systems). The tissue was allowed to settle on the MEA for at least 10 min before recording and continuously perfused with oxygenated Ringer's solution. During recordings, Ringer's solution was removed and odor stimulus (70 μL) was applied to the tissue using a pipette. Baseline spontaneous activity or stimulus-evoked activity in the OE was recorded for 3 min. After each 3-min recording, the tissue was rinsed at least three times with oxygenated Ringer's solution, and allowed to recover for 3 min before continuing with additional stimuli. Odors were dissolved as 10 mM stock solution in water and DMSO. Odor mix1 contained eugenol, geraniol, allyl phenylacetate, 1-octanol, benzyl acetate, (R)-(-)-carvone, 2-heptanone, and citral, while mix2 included octanoic acid, heptanoic acid, coumarin, linalool, octanal, β-citronellol, (S)-(+)-carvone, and trans-cinnamaldehyde. Single odorant included 2-heptanone, 1-octanol, citral, eugenol. In mammals, the OE is directly exposed to the external environment. Therefore, the temperature of the MEA culture chamber was maintained at 28 °C using the integrated heating element of the MEA2100 amplifier. Signals were amplified (×1200), digitized (1 kHz), filtered (< 500 Hz), stored, and exported as axon binary files for offline analyses with custom-written MATLAB (The MathWorks) scripts.

Raw signals were band-filtered (0.1-300 Hz) using zero-phase digital filter “filtfilt” to prevent phase distortion. In our previous studies, we demonstrated that odors mainly evoked slow oscillation (< 12 Hz) in the OE in vitro^19^. Therefore, we focused analysis on low-frequency local field potentials (LFPs, 0.1-12 Hz) in this study. Every 3 min, the signal was cut into non-overlapping 10-s-long segments. The band power of each segment was calculated using the “bandpower” function. Results were then averaged across all segments. The recorded LFP response was converted to ΔP, where ΔP was calculated by subtracting the averaged LFP (in the 0.3-12 Hz range) power before odor presentation from the averaged power during odor presentation. The power change was proportional to the change in amplitude of slow oscillations.

### scRNA-seq raw data processing

Raw sequencing data were processed using the 10x Genomics Cell Ranger pipeline (version 5.0.0). FASTQ files were aligned to the mouse reference genome (mm10), and the quality of sequencing was evaluated during alignment. Digital gene expression matrices were generated, with transcript expression levels quantified by unique molecular identifiers (UMIs). The filtered gene expression matrices were subsequently used for downstream analyses.

### scRNA-seq analysis

Human olfactory mucosa data from Allison D. Oliva *et al.*^20^ was downloaded from the NCBI Gene Expression Omnibus database (GEO GSE184117). scRNA-seq analysis for two OE samples from untreated and DOX-induced TRE-TNFα/Cyp2g1-rtTA mice was performed by BGI (Qingdao, China). All data were analyzed in R (version 4.2.2) using the Seurat package (version 5.2.0). For quality control of mice data, cells with fewer than 1,000 UMIs, fewer than 200 genes or more than 8,000 genes detected, or with > 10% mitochondrial transcripts were excluded. Genes expressed in fewer than 3 cells were also removed. After filtering, 63,199 cells (34,019 cells from untreated group and 29,180 cells from DOX group) were retained for analysis. Integration and normalization across samples were performed using the “IntegrateData” function in Seurat. Normalized UMI values for each cell were obtained with “NormalizeData” by scaling sequencing reads of each gene to total UMIs. Expression values were then scaled and centered using “ScaleData” for dimensionality reduction, which was conducted with “RunPCA.” Principal components (PCs) were used for subsequent dimensional reduction and clustering analyses.

For analyzing the reported human olfactory mucosa data, we excluded cells that expressed fewer than 200 genes and genes that were detected in fewer than three cells when reading the feature-barcode matrices into R (version 4.2.2). We used Seurat R package (version 5.2.0) to remove cells with over 10% mitochondrial reads. Similarly, cells with fewer than 100 or more than 8,000 features and fewer than 100 counts were filtered out. Integration of the datasets were performed using the "FindIntegrationAnchors" and "IntegrateData" functions. The integrated data was normalized using the "NormalizeData" function. The top 2,000 variable features were identified using "FindVariableFeatures" function with "vst" selection method.

### Cell type identification

Clustering was performed using the “FindNeighbors” and “FindClusters” functions with the first 60 PCs (determined by the elbow plot) and a resolution of 0.3 for mice data. Dimensional reduction was visualized with the “RunUMAP” function. Marker genes for each cluster were identified using the “FindAllMarkers” function with the MAST test implemented in Seurat. Cell type identities were assigned based on established marker genes. For analyses of mOSN subpopulations, normalized expression data were extracted from identified cell types using the “Subset” function.

For human data, total cell clustering was performed by "FindNeighbors" and "FindClusters" functions using the first 50 PCs and a resolution of 0.5. Dimensional reduction was performed with the “RunTSNE” function. For each cell type, we used multiple cell type-specific marker genes to determine cell-type identity. The visualization of marker expression is displayed through the “Dotplot” function in Seurat. To visualize DDIT3 expression in individual cells, expression values were extracted from the scRNA-seq data object and overlaid onto t-SNE embeddings using ggplot2. The expression of DDIT3 across different cell types was visualized using the "VlnPlot" function in Seurat.

### Identification of Ddit3 expression-related genes

The “FindMarkers” function in Seurat was used to identify differentially expressed genes (DEGs) in mOSNs between DOX and untreated group. Log fold change (Log2FC) and adjusted p values were calculated using the non-parametric two-sided Wilcoxon rank-sum test. DEGs were defined as |Log2FC| > 0.5 and adjusted p < 0.05. Subsequently, the DOX group within the mOSN subpopulation was further divided into Ddit3^+^ and Ddit3^-^ groups based on Ddit3 expression, and DEGs between these two groups were determined using the “FindMarkers” function.

### Gene ontology (GO) analysis

GO enrichment analysis was performed using the clusterProfiler R package (version 4.14.4, https://bioconductor.org/packages/clusterProfiler/) and visualized with ggplot2 (version 3.5.1, https://github.com/tidyverse/ggplot2). For multiple cell types, enrichment analysis was performed using the “compareCluster” function in clusterProfiler. A p value cutoff of 0.01 was applied. Representative GO terms from the top 25 ranked terms were displayed.

### SCENIC analysis

To infer transcription factor (TF) regulatory networks and assess regulon activity at the single-cell level, we applied the SCENIC workflow (version 1.3.1). Genes expressed in fewer than 3 cells and cells with fewer than 200 detected genes were excluded. To improve computational efficiency, the dataset was downsampled to 1,500 cells per group prior to analysis. The filtered expression matrix was used as input for SCENIC. Regulon activity scores (AUC, area under the curve) were calculated for each cell, and cell type-specific differences were visualized (e.g., violin plots for Ddit3). Regulons of interest were extracted using an AUC threshold of > 0.06. TF-target interactions were parsed from SCENIC output, filtered to remove redundant entries, and ranked by interaction weight. The top 100 high-confidence TF-target pairs were selected and exported as a tab-delimited file. These interactions were subsequently imported into Cytoscape for visualization of TF-target regulatory networks.

For human data, scRNA-seq expression matrix was log-normalized. The GENIE3 algorithm was used to construct co-expression networks between transcription factors and their target genes, followed by motif enrichment analysis using the RcisTarget human database (hgcn_v9) to identify regulons. The AUCell algorithm was applied to calculate regulon activity scores for individual cells. Regulon specificity scores (RSS) were computed using “calcRSS” function, and regulatory activity differences across cell types under different conditions were visualized through “plotRSS” function. To specifically visualize DDIT3-centered regulatory networks in mOSNs, we isolated the top 30 highest-confidence regulatory interactions (ranked by edge weight) where DDIT3 functions as the transcription factor regulating downstream target genes, based on GENIE3 co-expression network analysis. The network was constructed using the igraph package (version 2.0.3).

### Statistical analysis

GraphPad Prism (version 8) was used to perform statistical analysis, and all the data were showed as mean ± SEM. Statistical significance was analyzed using unpaired Student's t-test, one-way ANOVA with Dunnett's multiple comparisons test, one-way ANOVA with Tukey's multiple comparisons test, two-way ANOVA with Sidak's multiple comparisons test. The statistical significance was assigned as *p < 0.05, **p < 0.01, ***p < 0.001, and ****p< 0.0001, respectively.

## Results

### Characterization of two olfactory inflammation mouse models

We firstly characterized an inducible olfactory inflammation mouse model by doxycycline (DOX) induction and LPS-induced mouse inflammatory model. Using Cyp2g1-rtTA / TRE-TNFα mice, DOX treatment induced the generation of TNFα and created a chronic inflammatory microenvironment in the OE. Immunostaining data showed that DOX treatment reduced the number of OMP^+^ mature neurons by 28 ± 6% (p = 0.0034), but not IL33^+^ SUS cells in the OE (Fig. S1A). DOX also decreased the number of Ki67^+^ cells by 25 ± 5% (p = 0.0047), and increased the number of F4/80^+^ macrophages by 67 ± 15% (p = 0.0005) (Fig. S1B), indicating that chronic TNFα exposure impairs the neuronal maturation and enhances inflammation in the OE. We also performed intranasal instillation of LPS in WT mice to set up an olfactory inflammation model. Immunostaining data showed that the numbers of OMP^+^ mature neurons, IL33^+^ supporting cells, and Ki67^+^ proliferative cells were significantly decreased by 24 ± 4% (p = 0.0041), 23 ± 3% (p = 0.0016) and 62 ± 3% (p < 0.0001) in the OE of LPS-induced inflammation model compared to saline control, while the numbers of CD45^+^ inflammatory cells and F4/80^+^ macrophages were drastically increased by 218 ± 39% (p = 0.0004) and 54 ± 16% (p = 0.0147), indicating that LPS deteriorates cell proliferation, impairs sustentacular and neuronal differentiation, and aggravates inflammation in the OE (Fig. S2). The loss of sustentacular cells in the OE by LPS instillation was also supported by reduced number of apical Sox2^+^ cells (Fig. S3). This loss occurred in the dorsal, medial, and ventral but not in lateral OE (Fig. S3B-F). Collectively, we identify features in the OE of inducible TNFα mouse and LPS-instilled mouse models.

### OE organoid model for inducible olfactory inflammation

**Figure 1:**
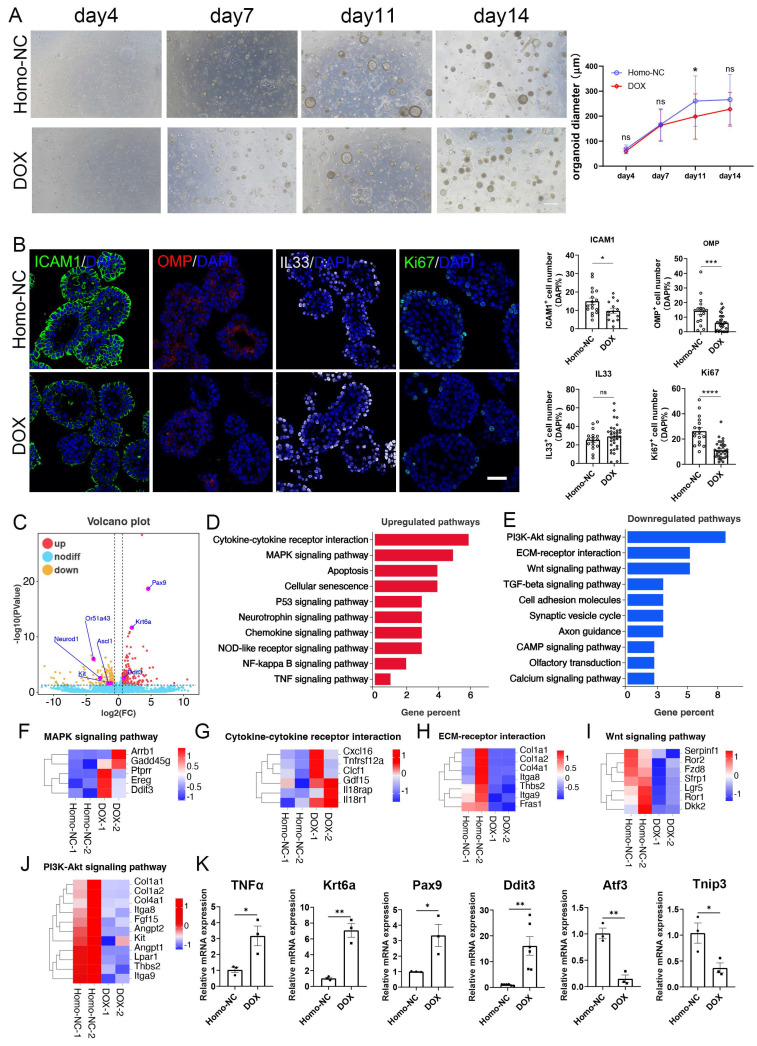
An inducible olfactory inflammation model based on OE organoids by Dox treatment. (A) Microscopic images of saline or Dox-treated OE organoids derived from Cyp2g1-rtTA/ TRE-TNFα mice, and quantification of organoid size with saline or Dox treatment. n= 36 organoids in each group. (B) Confocal images and quantification of ICAM1^+^, OMP^+^, IL33^+^, Ki67^+^ cells in saline and Dox-treated OE organoids from inducible olfactory inflammation mice. ICAM1^+^: n=17 and 14 organoids in control and DOX group, OMP^+^: n=17 and 29 organoids, IL33^+^: n=16 and 32 organoids, Ki67^+^: n=17 and 33 organoids. (C) Volcano plot showing upregulated and downregulated genes in Dox-treated inducible olfactory inflammation organoids. (D, E) Upregulated (D) and downregulated (E) pathways in Dox-treated OE organoids. (F-J) Heatmap plots of genes involved in MAPK signaling (F), cytokine receptor interaction (G), ECM-receptor interaction (H), Wnt signaling pathway (I), PI3K-Akt signaling pathway (J). (K) Quantitative PCR data showing the differential expression of representative genes in Dox-treated organoids compared to saline control. n=6 preparations for Ddit3, n= 3 preparations for others. The statistical significances were determined by two-way ANOVA with Sidak's multiple comparisons test in (A) and by unpaired t test in (B) and (K). ns, not significant, *p < 0.05, **p < 0.01, ***p < 0.001, **** p < 0.0001. Scales bars: 200 μm in (A), 20 μm in (B).

We then established an inducible olfactory inflammation organoid model by DOX induction. DOX-treated OE organoids derived from Cyp2g1-rtTA / TRE-TNFα mice showed reduction in size by 24 ± 6% (p = 0.0013) and 14 ± 4% (p = 0.0941) at Day 11 and Day 14 post culture, respectively, compared to untreated control (Fig. 1A). DOX-treated organoids showed decreased number of OMP^+^ mature neurons and Ki67^+^ proliferative cells by 56 ± 7% (p = 0.0007) and 57 ± 4% (p < 0.0001), respectively, while the number of IL33^+^ cells was not significantly changed (Fig. 1B). RNA-syeq data showed transcriptional alteration in DOX-treated organoids, such as upregulation of Ddit3, Pax9 and Krt6a, and downregulation of neuronal progenitor markers NeuroD1, Ascl1, and Kit (Fig. 1C). KEGG enrichment analysis showed upregulation in cytokine receptor interaction, MAPK signaling pathway, apoptosis, and cellular senescence, while downregulated genes mainly participated in PI3K-Akt and Wnt signaling pathways (Fig. 1D, E). Heatmap and quantitative PCR data confirmed the upregulation of TNFα, Ddit3, Pax9, and Krt6a, and downregulation of Atf3 and Tnip3 in DOX-treated organoids (Fig. 1F-K). Thus, an inducible inflammation in OE organoids deteriorates the neuronal differentiation and cell proliferation, providing an *in vitro* model mimicking chronic olfactory inflammation.

### Establishment of OE organoid model reflecting LPS-induced olfactory inflammation

**Figure 2:**
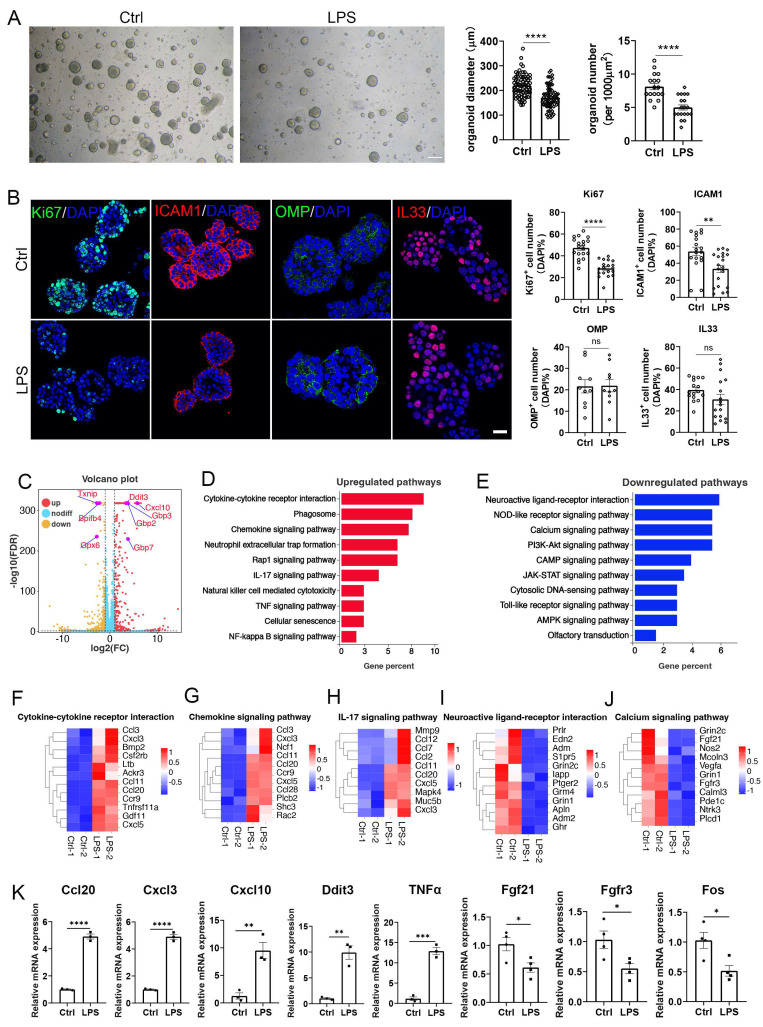
An inflammatory OE organoid model based on LPS instillation. (A) Microscopic images of OE organoids derived from saline or LPS-instilled mice, and the quantification of organoid diameter (n=72 and 74 organoids for control and LPS group) and number (n=17 and 19 preparations) in control and LPS groups. (B) Confocal images and quantification of Ki67^+^, ICAM1^+^, OMP^+^, and IL33^+^ cells in OE organoids derived from saline or LPS-instilled mice. Ki67^+^: n=19 and 19 organoids in Ctrl and LPS group, ICAM1^+^: n=18 and 20 organoids, OMP^+^: n=10 and 10 organoids, IL33^+^: n=16 and 17 organoids. (C) Volcano plot showing upregulated (red) and downregulated (orange) genes in organoids from LPS-instilled tissues compared to saline control. n=3 samples in each group. (D, E) KEGG enrichment analysis showing upregulated (D) and downregulated (E) pathways in OE organoids from LPS group compared to saline control. (F-J) Heatmap plots showing differentially expressed genes involved in cytokine receptor interaction, chemokine signaling pathway, IL-17 signaling pathway, neuroactive ligand-receptor interaction, and calcium signaling pathway. (K) Quantitative PCR analysis showing differential expression of representative genes between saline and LPS-instilled group. n=3 or 4 preparations. The statistical significances were determined by unpaired t test. ns, not significant, *p < 0.05, **p < 0.01, ***p < 0.001, **** p < 0.0001. Scales bars: 200 μm in (A), 20 μm in (B).

Then, we cultured OE organoids from LPS-instilled mice. Compared to saline control, organoids from LPS instillation model showed reduced size and number by 22 ± 2% (p < 0.0001) and 38 ± 5% (p < 0.0001) (Fig. 2A), suggesting that LPS impairs OE organoid growth. Organoids from LPS-instilled mice exhibited reduced numbers of Ki67^+^ cells and ICAM1^+^ cells by 39 ± 4% (p < 0.0001) and 38 ± 9% (p = 0.0035), respectively, showing that OE organoid model demonstrates impaired proliferation and stem cell maintenance by LPS intranasal instillation (Fig. 2B). However, no significant difference in the number of OMP^+^ or IL33^+^ cells was observed in this OE organoid model without continuous LPS treatment *in vitro* (Fig. 2B), suggesting that deficits in the sustentacular and neuronal differentiation by LPS instillation are not fully mimicked in organoid model without direct LPS addition in culture condition. RNA-seq analysis showed apparent transcriptional change in organoids derived from LPS model versus saline control, such as upregulation of Ddit3 and Cxcl10, as well as downregulation of Txnip and Bpifb4 (Fig. 2C). KEGG enrichment analysis indicated upregulated inflammation-related pathways such as cytokine receptor interaction, chemokine signaling, neutrophil extracellular trap (NET) formation, and IL17 signaling pathway, while downregulated genes were mainly participated in neuroactive ligand-receptor interaction, calcium signaling pathway and PI3K-Akt signaling pathway (Fig. 2D, E). Heatmap data from RNA-seq analysis and quantitative PCR analysis confirmed expression change of several critical molecules, such as upregulation of inflammation-related genes Ccl20, Cxcl3, Cxcl10, Ddit3, TNFα, and downregulation of cell proliferation and activation factors Fgf21, Fgfr3 and Fos (Fig. 2F-K). To further explain how LPS affected OE organoids, we continuously treated organoids with LPS *in vitro*. We found that the numbers of Ki67^+^ proliferative cells, OMP^+^ mature OSNs, IL33^+^ sustentacular cells, and ICAM1^+^ HBCs were significantly decreased by 56 ± 8% (p < 0.0001), 51 ± 8% (p = 0.0007), 56 ± 7% (p = 0.0011), and 36 ± 7% (p = 0.0066), respectively in LPS-treated organoids compared to saline controls (Fig. S4), suggesting the consistency between LPS-instilled OE tissues and LPS-treated OE organoids. Collectively, we establish an LPS-induced inflammatory OE organoid model, with apparent attenuation in cell proliferation and neuronal differentiation, and transcriptional upregulation of inflammation-related pathway.

### An inflammatory OE organoid model by TNFα treatment

**Figure 3:**
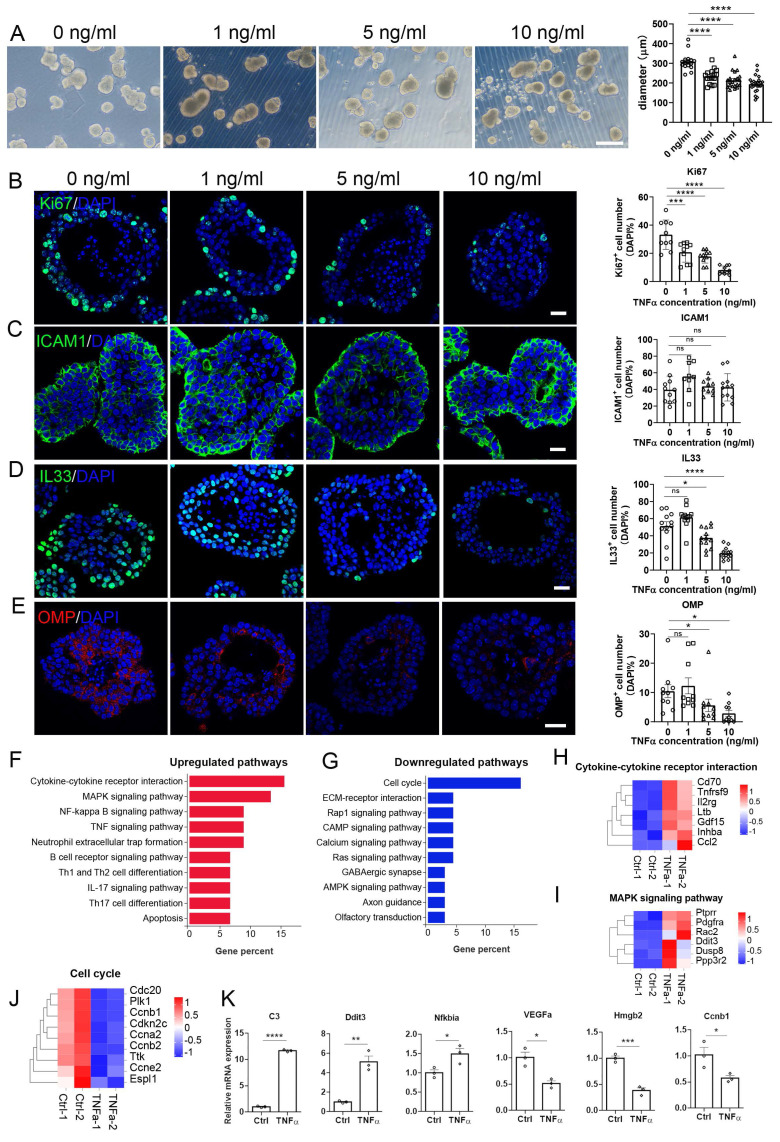
Establishment of inflammatory OE organoid model by TNFα treatment. (A) Microscopic images of OE organoids treated with different concentration of TNFα. n=16, 16, 21, 22 organoids with 0, 1, 5, 10 ng/ml TNFα. (B-E) Confocal images and quantification of Ki67^+^ (B), ICAM1^+^ (C), IL33^+^ (D), OMP^+^ (E) cells in TNFα-treated OE organoids. Ki67^+^: n = 10 organoids in each group, ICAM1^+^: n=11, 9, 10, 12 organoids, IL33^+^: n= 12, 13, 13, 13 organoids, OMP^+^: n = 10 organoids in each group. (F, G) KEGG enrichment analysis showing upregulated (F) and downregulated (G) pathways in OE organoids treated with 10 ng/ml TNFα compared to saline control. (H-J) Heatmap plots showing differentially expressed genes related with cytokine receptor interaction (H), MAPK signaling pathway (I), and cell cycle (J) in organoids treated with 10 ng/ml TNFα. (K) Quantitative PCR data confirming differential expression of representative genes in TNFα-treated OE organoids compared to saline control. n= 3 preparations. The statistical significances were determined by one-way ANOVA with Dunnett's multiple comparisons test in (B) and by unpaired t test in (K). ns, not significant, *p < 0.05, **p < 0.01, ***p < 0.001, **** p < 0.0001. Scales bars: 200 μm in (A), 20 μm in (B).

Next, we constructed TNFα-induced inflammatory OE organoid model. Organoids from WT OE were cultured and treated with different concentration of TNFα. We did not observe significant change in the number of Ki67^+^ or OMP^+^ cells at 24 or 48 h post TNFα treatment (Figs. S5 and S6). At 72 h post treatment, the organoid size was significantly decreased by 24 ± 3%, 30 ± 3%, and 37 ± 3% when treated with 1, 5 and 10 ng/ml TNFα, respectively (Fig. 3A, p < 0.0001).

The number of Ki67^+^ cells were reduced by 37 ± 7% (p = 0.0007), 46 ± 4% (p < 0.0001) and 75 ± 3% (p < 0.0001) when treated with 1, 5 and 10 ng/ml TNFα, respectively (Fig. 3B). Besides, the number of IL33^+^ cells was reduced by 23 ± 6% (p = 0.0213) and 63 ± 4% (p < 0.0001) at 5 and 10 ng/ml TNFα, respectively (Fig. 3D), and the number of OMP^+^ mature neurons was decreased by 66 ± 7% (p = 0.0447) and 72 ± 10% (p = 0.0205) when treated with 5 and 10 ng/ml TNFα, respectively (Fig. 3E). RNA-seq data showed that upregulated genes in TNFα-treated OE organoids were mainly involved in cytokine receptor interaction, NF-κB signaling pathway and TNF signaling pathway, while downregulated genes were predominantly associated with cell cycle (Fig. 3F, G). Heatmap data combined with qPCR analysis validated the upregulation of C3, Ddit3, and NFkbia, as well as downregulation of a few cell-cycle-related genes such as Hmgb2 and Ccnb1 in TNFα-treated organoids (Fig. 3H-K). Above all, these data show the establishment of an inflammatory model based on TNFα-treated OE organoids, exhibiting impaired sustentacular and neuronal differentiation, enhanced inflammation and attenuated cell cycle.

### Ddit3 downregulation alleviates cell proliferation and neuronal differentiation in inflammatory OE organoid model

**Figure 4:**
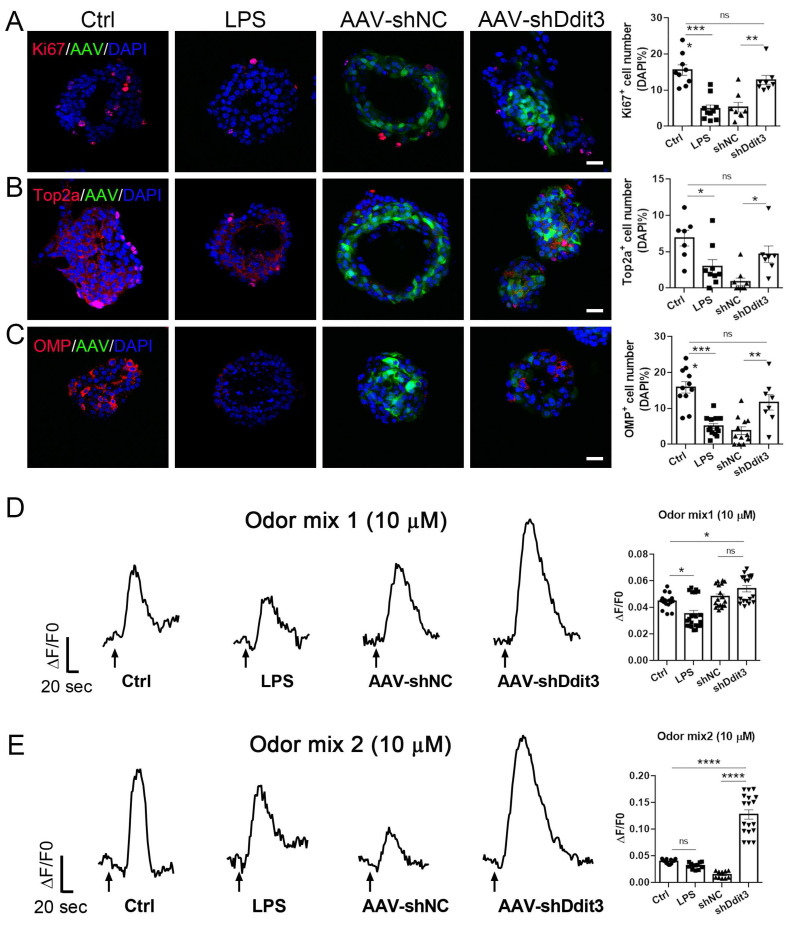
Ddit3 downregulation counteracts the effect of LPS in OE organoids. (A-C) Confocal images and quantification of Ki67^+^ (A), Top2a^+^ (B), OMP^+^ (C) cells in OE organoids treated with saline or LPS, and in LPS-treated organoids infected with control AAV (AAV-shNC) or AAV expressing shRNA targeting Ddit3 (AAV-shDdit3). Ki67^+^: n= 9, 10, 8, 8 organoids in control, LPS, LPS+shNC, LPS+shDdit3 group, Top2a^+^: n= 7, 9, 9, 7 organoids, OMP^+^: n= 11, 13, 12, 8 organoids. (D, E) Representative calcium imaging curves of OE organoids treated with saline or LPS, and in LPS-treated organoids infected with AAV-shNC or AAV-shDdit3, simulated with odor mix1 (D) and mix2 (E). Quantifications of calcium imaging data were shown at the right. Mix1: n= 16, 21, 18, 18 recordings in control, LPS, LPS+shNC, LPS+shDdit3 group, Mix2: n= 15, 12, 10, 18 recordings. The statistical significances were determined by one-way ANOVA with Tukey's multiple comparisons test. ns, not significant, *p < 0.05, **p < 0.01, ***p < 0.001, **** p < 0.0001. Scales bars: 20 μm.

Our RNA-seq and quantitative data showed the upregulation of Ddit3 at transcriptional level in inflammatory OE organoids. We then found that the number of Ddit3^+^ cells was significantly increased by 89 ± 16% (p = 0.0003), 71 ± 17% (p = 0.0073), and 107 ± 19% (p = 0.0003) in LPS, TNFα, and DOX induction-based inflammatory OE organoids, respectively, indicating the increase in Ddit3 expression at protein level (Fig. S7A-C). To further support this finding, Western blot data showed the Ddit3 upregulation in LPS- or DOX-induced olfactory inflammation mouse model (Fig. S8A-D), and LPS- or TNFα-treated OE organoids (Fig. S8E-H), when compared to their respective control counterpart. To elucidate the function of Ddit3, we downregulated Ddit3 expression by AAV-shRNA infection in OE organoids, showing 69±3% and 82±1% decrease in Ddit3 expression level when infected with 10^8^ and 10^10^ AAV titers (Fig. S7D, p < 0.0001). In OE organoids treated with LPS, Ddit3 downregulation increased the numbers of Ki67^+^ and Top2a^+^ cells by 143 ± 24% (p = 0.0021) and 441 ± 132% (p = 0.0346), respectively, compared to AAV-shNC group (Fig. 4A, B). Furthermore, the numbers of Ki67^+^ and Top2a^+^ cells in AAV-shDdit3 group were comparable to LPS-untreated group, suggesting Ddit3 downregulation recovers cell proliferation to normal level in LPS-induced inflammatory OE organoid model (Fig. 4A, B; Ki67^+^: p = 0.4486; Top2a^+^: p = 0.3925). Besides, the number of OMP^+^ mature neurons in LPS-treated organoids infected with AAV-shDdit3 was increased by 208 ± 56% (p = 0.0018) compared to AAV-shNC group, while this number in Ddit3 downregulation groups were not significantly different from organoids without LPS exposure (p = 0.1855, Fig. 4C). Furthermore, calcium imaging analysis showed that Ddit3 downregulation enhanced response to two odor mixes in LPS-treated organoids, compared to AAV-shNC control group (Fig. 4D, E, mix1: p = 0.2443, mix2: p < 0.0001). These data suggest that Ddit3 downregulation alleviates cell proliferation and generation of mature sensory neurons, and recovers functional response to odors in LPS-induced inflammatory organoid model.

Ddit3 downregulation also recovered cell proliferation in other two inflammatory OE organoid models, with increase by 122 ± 21% (p = 0.0002) and 177 ± 43% (p = 0.0152) in the number of Ki67^+^ cells, and by 149 ± 21% (p = 0.0002) and 342 ± 77% (p = 0.0009) in the number of Top2a^+^ cells in TNFα and DOX-treated organoids infected with AAV-shDdit3, respectively, compared to AAV-shNC group (Figs. S9A, B and S10A, B). Ddit3 downregulation also increased the number of OMP^+^ mature neurons by 66 ± 21% (p = 0.0905) and 95 ± 27% (p = 0.0493) in TNFα-treated and DOX-induced OE organoids, respectively, when compared to AAV-shNC-infected organoids (Figs. S9C and S10C). More importantly, the number of OMP^+^ neurons in AAV-shDdit3-infected organoids treated with TNFα or DOX was comparable to respective untreated normal controls (p = 0.4257 and p = 0.5133, Figs. S9C and S10C), suggesting that generation of mature sensory neurons in these two inflammatory organoid models was recovered by Ddit3 downregulation. The response to odor mixes was enhanced in TNFα or DOX-treated organoids infected with AAV-shDdit3, compared to respective AAV-shNC group (TNFα: p = 0.0039 for mix1, p < 0.0001 for mix2; DOX: p < 0.0001 for mix 1 and 2, Figs. S9D, E and S10D, E). Furthermore, LPS treatment also reduced responses to single odorant in OE organoids (p < 0.0001 for each odorant), while Ddit3 downregulation recovers these responses to 2-hepatanone, 1-octanol, citral, eugenol when compared to respective AAV-shNC group (p < 0.0001 for each odorant, Fig. S11).

We also found that the ratio of TUNEL^+^ cells were increased by 2.7 ± 0.4 folds, 2.8 ± 0.4 folds, and 5.9 ± 0.7 folds in DOX-induced, LPS-treated, and TNFα-treated organoids, respectively, compared to controls (Fig. S12A-F, p < 0.0001 for each group). Moreover, Ddit3 downregulation by AAV-shDdit3 infection reduced the ratio of TUNEL^+^ cells by 58 ± 3% (p < 0.0001), 50 ± 5% (p = 0.003), 67 ± 3% (p < 0.0001) in DOX-induced, LPS-treated, and TNFα-treated organoids, respectively, compared to AAV-shNC groups (Fig. S12A-F). Furthermore, the ratio of TUNEL^+^ cells in inflammatory organoids infected with AAV-shDdit3 was not significantly different from untreated normal control group (DOX: p = 0.7094, LPS: p = 0.9771, TNFα: p = 0.6173). Therefore, Ddit3 downregulation alleviates apoptosis that was exacerbated by inflammation in OE organoids, recovering to the threshold of untreated normal controls. Collectively, we conclude a beneficial role of Ddit3 downregulation in reducing the apoptosis, recovering cell proliferation, sensory neuronal differentiation, and functional response in olfactory inflammation organoid models.

### Ddit3 deficiency counteracts the effect of LPS in the OE

**Figure 5:**
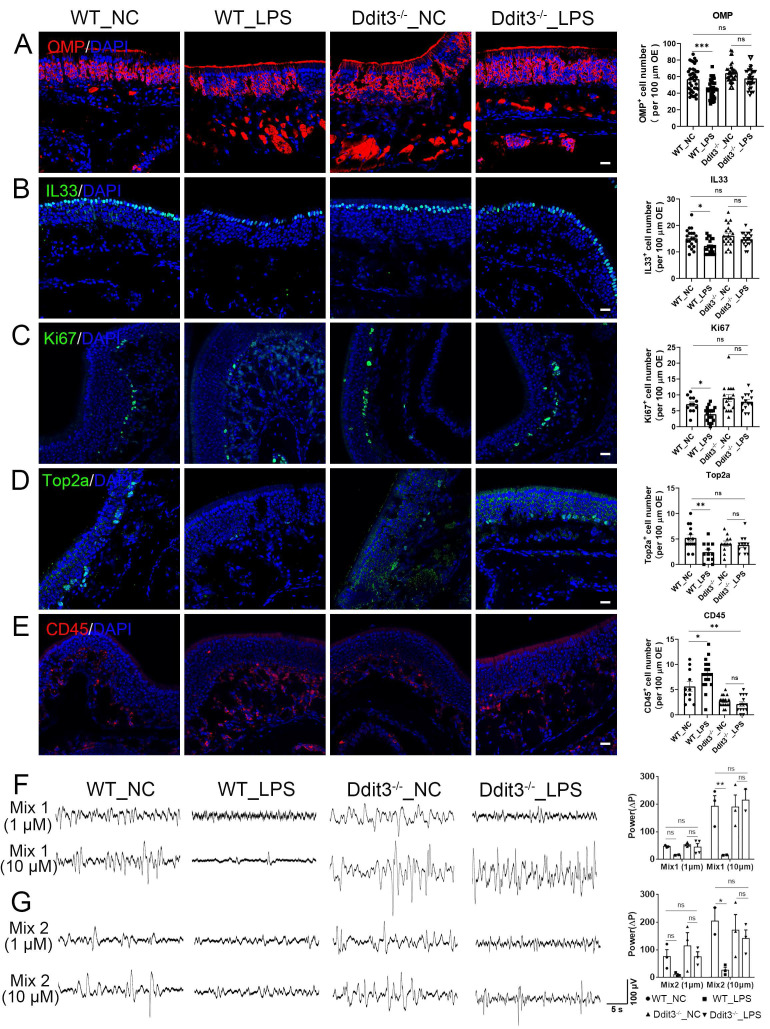
Ddit3 deficiency counteracts the effect of LPS instillation in the OE. (A-E) Confocal images and quantification of OMP^+^ (A), IL33^+^ (B), Ki67^+^ (C), Top2a^+^ (D), CD45^+^ (E) cells in the OE of saline or LPS-instilled WT mice, and saline or LPS-instilled Ddit3^-/-^ mice. OMP^+^: n= 39, 40, 40, 40 OE regions in WT_NC, WT_LPS, Ddit3^-/-^_NC, Ddit3^-/-^_LPS group, IL33^+^: n= 20 OE regions in each group, Ki67^+^: n= 12, 18, 17, 14 OE regions, Top2a^+^: n= 14, 12, 11, 12 OE regions, CD45^+^: n= 11, 18, 17, 14 OE regions. (F, G) The local field potential (LFP) of the OSNs to odor mix1 (F) and mix2 (G) in OE of WT and Ddit3^-/-^ mice instilled with saline or LPS. Quantification of the ΔP of LFP (ΔP = P_odor mix_ - P_baseline_) was shown on right. Mix1 (1µm): n= 3, 2, 4, 4 OE tissues in WT_NC, WT_LPS, Ddit3^-/-^_NC, Ddit3^-/-^_LPS group, Mix1 (10µm): n= 3, 2, 3, 2 OE tissues, Mix2 (1µm): n= 3 OE tissues in each group, Mix2 (10µm): n= 2, 3, 3, 3 OE tissues. The statistical significances were determined by one-way ANOVA with Tukey's multiple comparisons test in (A-E), and by two-way ANOVA with Tukey's multiple comparisons test in (F, G). ns, not significant, *p < 0.05, **p < 0.01, ***p < 0.001. Scales bars: 20 μm.

With LPS instillation, Ddit3 was upregulated in the OE compared to control group at protein level (Fig. S8E, F). Since LPS instillation reduced the number of mature neurons, supporting cells, and proliferative cells, and increased the number of inflammatory cells in the OE, we determined whether Ddit3 deficiency could counteract this process. In Ddit3^-/-^ OE (Fig. S13A-C), LPS instillation did not significantly change the number of OMP^+^ mature neurons (p = 0.1095), IL33^+^ supporting cells (p = 0.5666), Ki67^+^ (p = 0.7428) and Top2a^+^ proliferative cells (p = 0.9886) when compared to the OE of LPS-free Ddit3^-/-^ mice, while the numbers of these cell types showed significant reduction in LPS-instilled WT mice than untreated WT control (Fig. 5A-D). Moreover, the numbers of these cells in Ddit3^-/-^ OE with LPS instillation were comparable to those in saline-treated WT mice (OMP^+^: p = 0.9864, IL33^+^: p = 0.9916, Ki67^+^: p = 0.9223, Top2a: p = 0.2786), further supporting the alleviation in homeostasis of supporting cells and sensory neurons, as well as cell proliferation in LPS-impaired OE by Ddit3 deletion. We also found that the number of CD45^+^ inflammatory cells was not significantly different between saline control and LPS group in Ddit3^-/-^ mice (p = 0.908), while LPS instillation significantly increased the number of CD45^+^ cells in WT mice (Fig. 5E). These results suggest that LPS does not induce inflammation in Ddit3^-/-^ OE. Compared to WT mice with or without LPS instillation, Ddit3 deletion significantly reduced the number of CD45^+^ cells in the OE (Fig. 5E, untreated: p = 0.0102, LPS: p < 0.0001), suggesting that Ddit3 deletion apparently inhibits inflammation. Moreover, LPS instillation increased the number of apoptotic cells by 2.9 ± 0.6 folds (p = 0.0001) in the OE, while LPS did not lead to significant increase in the number of apoptotic cells in Ddit3^-/-^ mice (p = 0.9748, Fig. S12G, H). This showed that Ddit3 deficiency inhibits LPS-induced apoptosis in the OE.

We then asked whether Ddit3 deficiency recovered olfactory function in LPS-instilled mice by acutely isolating the OE and performing *in vitro* electrophysiological recordings (Fig. S13D). The responses to odor mixes at two different concentrations were quantified by the relative power (ΔP). Compared to saline-instilled Ddit3^-/-^ mice, LPS treatment showed non-significant change in ΔP when treated with odor mixes at 10 μM (Fig. 5F, G, p = 0.9121 for Mix 1, p = 0.9182 for Mix 2), while LPS instillation significantly reduced the relative power to 10 μM odor mixes in the WT OE (Fig. 5F, G, p = 0.0013 for Mix 1, p = 0.0196 for Mix 2). Furthermore, stimulation with single odorant also elicited electrophysiological response in the OE (Fig. S13E, F), and showed the similar changes as the odor mix, with relative power reduction by LPS instillation and recovery in LPS-instilled Ddit3^-/-^ mice (Fig. S13G, H). These data suggest a protective role of Ddit3 deficiency for functional response against LPS-induced olfactory inflammation. Collectively, we conclude that Ddit3 deficiency counteracts the adverse effect of LPS on sustentacular and neuronal homeostasis, cell proliferation, inflammatory activation, and functional response to odors in the OE.

### Ddit3 upregulation in olfactory inflammation model correlates with ER stress and apoptotic process

**Figure 6:**
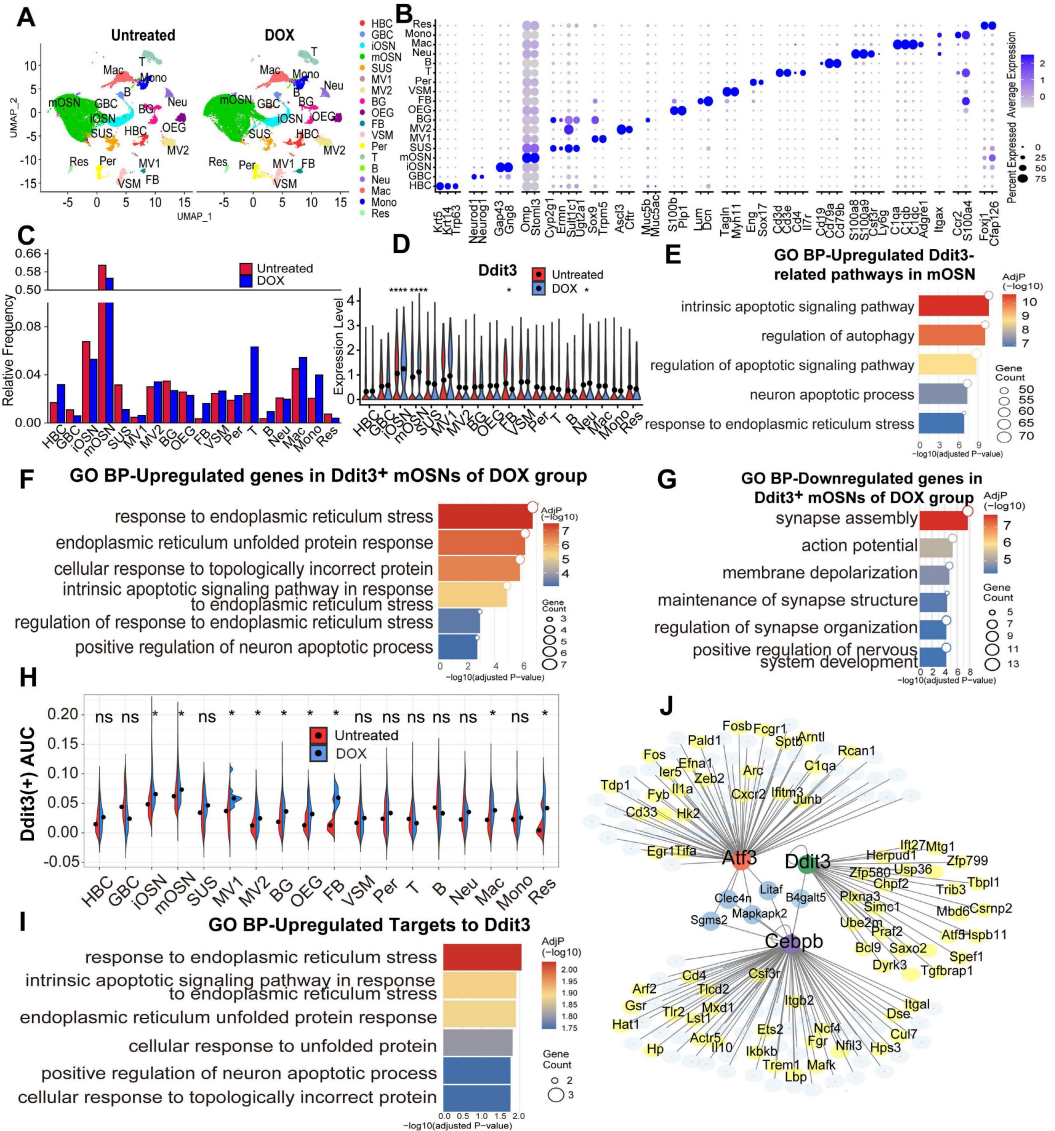
Ddit3 upregulation in mOSN of inducible olfactory inflammation model correlates with ER stress and apoptotic process. (A) UMAP plot showing 18 cell types of OE from Cyp2g1-rtTA/ TRE-TNFα mice with or without DOX treatment. n=2 samples in each group. (B) Dot plot showing the expression of representative molecular markers for each cell type. (C) Bar graph showing the proportion of all cell types in the OE of untreated and DOX groups. (D) Violin plot of differential Ddit3 expression level in all OE cell types between untreated and DOX group. (E-G) GO enrichment analysis on upregulated Ddit3-related genes in mOSNs from inflammatory model (E), upregulated (F) and downregulated (G) genes in Ddit3^+^ mOSNs of DOX group. (H) Activity scores of Ddit3 regulon in all OE cell types of untreated and DOX group. (I) SCENIC analysis showing the transcriptional network on mOSNs in inflammatory model. (J) GO terms of upregulated target genes to Ddit3. Statistical significance was determined by Wilcoxon rank sum test in (D), and by t test in (H). ns, not significant, * p < 0.05, **** p < 0.0001.

To elucidate the role of Ddit3 in olfactory inflammation, OE tissues derived from DOX-treated and -untreated Cyp2g1-rtTA/TRE-TNFα mice were subjected to scRNA-seq analysis. We identified 18 cell types based on the expression matrix of their specific markers (Fig. 6A, B). The percentages of GBC, iOSN, mOSN, and sustentacular (SUS) cells were reduced in the OE of DOX-treated mice, while the ratio of inflammatory and immune cells such as T cell, B cell, macrophage, monocyte was increased (Fig. 6C). Ddit3 was upregulated in iOSN, mOSN and neutrophil of DOX-treated group compared to untreated control (Fig. 6D). Ddit3-related GO terms upregulated in mature sensory neurons of DOX-treated mice included apoptotic signaling pathway, neuron apoptotic process, and response to endoplasmic reticulum (ER) stress (Fig. 6E).

In DOX-induced olfactory inflammation model, upregulated genes expressed in Ddit3^+^ mOSNs compared to Ddit3^-^ neurons mainly functioned in response to ER stress, unfolded protein response, and positive regulation of neuron apoptotic process (Fig. 6F), while downregulated genes were correlated with a series of neural activity such as synapse assembly, action potential, and membrane depolarization (Fig. 6G). Activity scores of Ddit3 regulon were higher in iOSN and mOSN of DOX-treated inducible inflammation model compared to untreated group (Fig. 6H). SCENIC analysis showed that Ddit3 is a hub in the transcriptional network and regulated a subset of downstream target genes (Fig. 6I). These upregulated target genes functioned in response to ER stress, unfolded protein response, and positive regulation of neuron apoptotic process (Fig. 6J). Collectively, these data show that Ddit3 upregulation in mature OSNs of olfactory inflammation model potentially regulates ER stress and apoptotic process.

### DDIT3 correlates with ER stress and intrinsic apoptosis in mature OSNs of presbyosmic patients

**Figure 7:**
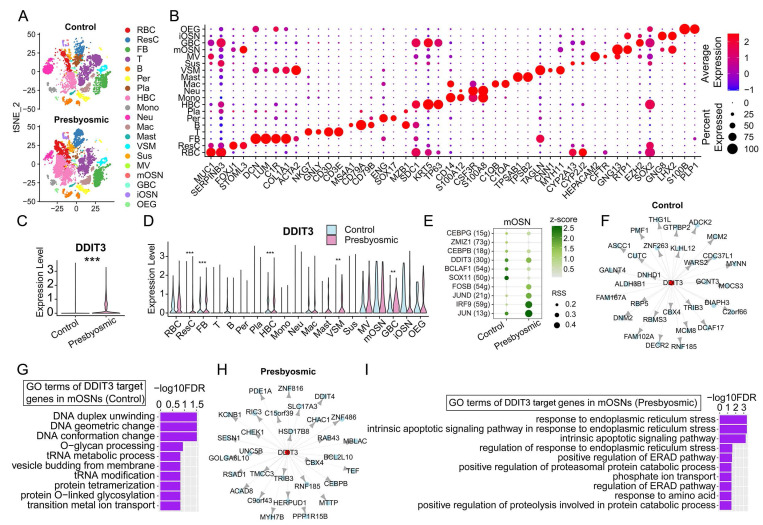
DDIT3 correlates with ER stress and intrinsic apoptosis in presbyosmic patients. (A) tSNE plot showing 19 cell types in olfactory mucosa from controls with normosmic smell functions and patients with aging-related olfactory loss. n= 3 samples in each group. (B) Dot plot showing the expression of representative molecular markers for each cell type. (C, D) Violin plot showing DDIT3 expression in olfactory mucosa of control and presbyosmic patients (C), and in each type (D). (E) The regulon activity in mature OSNs from control and presbyosmic patients by SCENIC analysis. RSS indicated intensity and z-score represented specificity. (F, H) Network visualization depicts the top 30 high-confidence regulatory interactions in mature OSNs from controls (F) and presbyosmic patients (H) predicted by GENIE3, where DDIT3 functions as a transcription factor regulating downstream target genes. (G, I) Top 10 GO terms of DDIT3 target genes in mature OSNs from controls (G) and presbyosmic patients (I). Statistical significance was determined by Wilcoxon rank sum test. ** p < 0.01, *** p < 0.001.

We then determined if DDIT3 correlated with response to ER stress and apoptotic pathway in patients with aging-related olfactory dysfunction. We analyzed scRNA-seq data of olfactory mucosa from control with normosmic smell function and presbyosmic patients reported by Allison D. Oliva *et al.* (GEO GSE184117)^20^. We identified 19 cell types in olfactory mucosa tissues based on the expression matrix of their specific markers (Fig. 7A, B). Overall, DDIT3 was significantly upregulated in olfactory mucosa of patients with aging-related olfactory dysfunction compared to controls with normosmic smell function (Fig. 7C). Significant upregulation of DDIT3 was observed in several cell types including GBC and HBC, but not in mature OSN (Fig. 7D). SCENIC analysis showed that DDIT3 regulon exhibited higher specificity score of transcriptional activity in mature OSNs of patients than normal controls (Fig. 7E). GENIE3 predicted the regulatory interaction where DDIT3 functioned as a hub to regulate downstream target genes, mainly associated with DNA conformation change in mOSNs of normosmic controls (Fig. 7F, G). By contrast, DDIT3 regulon functioned in response to ER stress and intrinsic apoptotic signaling pathways in mOSNs of patients with aging-related olfactory dysfunction (Fig. 7H, I). Collectively, these data support that DDIT3 expression in mature OSNs of presbyosmic patients correlates with ER stress and induced intrinsic apoptosis.

## Discussion

In this study, we established three types of olfactory inflammation organoid models. Ddit3 is a target gene candidate that upregulated in inflammatory models. Downregulation of Ddit3 counteracted apoptosis, improved cell proliferation, neuronal differentiation, and odor response in olfactory inflammation organoids. Ddit3 deficiency recovered cell proliferation, neuronal and sustentacular cell homeostasis, and functional response to odor stimulation. These results suggest a potential intervention of Ddit3 in inflammation-related olfactory dysfunction, while established olfactory inflammation organoid models may function as tools to screen potential drug and therapeutic target to olfactory disorders.

Chronic inflammation is one of major factors to olfactory dysfunction. Until now, several chronic olfactory inflammation models have been established to study the consequences of inflammation on the olfactory system, such as intranasal administration of LPS^7^, and induction of TNFα using DOX-controlled transgenic mice^4^, ^21^. However, an effective *in vitro* model to mimic the olfactory loss and to explain its pathogenesis is lacking. Here, we made use of our established 3D-cultured OE organoids^9^, to construct olfactory inflammation models. This OE organoid has been used to reveal and validate the function of critical genes in OE homeostasis and regeneration^12^-^16^. The current work extends its application to study the effect of inflammation on olfactory epithelial cell homeostasis and olfactory function *in vitro*. These three organoid models mimic phenotypes of olfactory inflammatory animal models, showing increased apoptosis, reduced cell proliferation and neuronal generation, as well as weaker functional response to odors. These organoid models were also capable of screening the candidates to counteract the outcomes by inflammatory stimulation. Thus, these olfactory inflammation organoid models offset shortcomings of animal models, providing easier accessibility and manipulation.

Ddit3 (also known as CHOP) is a transcriptional factor related to ER stress that causes changes in gene expression that favor apoptosis^22^. It was reported that suppression of Ddit3 reduced apoptosis in hippocampal neurons and rescues cognitive impairment^23^, while Ddit3 upregulation was correlated with photoreceptor cell degeneration^24^ and follicular atresia^25^ via ER stress activation. These regulatory effects of Ddit3 expression level were consistent with our finding that Ddit3 was upregulated in olfactory inflammation organoid models, while downregulation or deficiency of Ddit3 rescued cell proliferation, neuronal maturation and functional response to odors in inflammatory organoid and mouse models (Figs. 4, 5, S9-11, S13). In OSNs, unfolded protein response transcripts including Ddit3 were upregulated by application of olfactotoxic chemical methimazole^26^. Upregulation of Ddit3 was also found in olfactory bulb with infusion of Tunicamycin, leading to ER stress^27^. These reports support our scRNA-seq data of mature OSNs in inducible olfactory inflammation model that upregulation of Ddit3 associates with upregulating ER stress, unfolded protein response, and subsequent apoptotic process, as well as downregulating neural activity (Fig. 6). Combining with our findings that Ddit3 is upregulated and correlates with cell damage and apoptosis in olfactory inflammation model, we conclude that Ddit3 is an important regulator in response to injury in olfactory system. In patients with aging-related olfactory dysfunction, DDIT3 may regulate response to ER stress and intrinsic apoptotic pathway (Fig. 7). This further shows a potential application for Ddit3 as a therapeutic target to olfactory dysfunction.

## Conclusions

We established olfactory inflammation organoid models, and used these models to identify Ddit3 as a potential therapeutic target against olfactory inflammation and neuronal loss to recover olfactory function.

## Supplementary Material

Supplementary figures.

## Data Availability

The datasets generated and/or analysed during the current study are available in the China National GeneBank DataBase (CNGBdb) repository (CSE0000541).
