# Supplementary material for: Olfactory epithelium organoid models identify Ddit3 as a potential therapeutic target against inflammation-related olfactory sensory neuronal loss and functional deficit
Source: Int J Biol Sci. 2026 Mar 30;22(8):4025–42. doi: 10.7150/ijbs.129192 (PMC13137857; doi:10.7150/ijbs.129192)
Supplement: Supplementary file 1 — Supplementary figures. [file ijbsv22p4025s1.pdf]

Supplementary figures for

# Olfactory epithelium organoid models identify Ddit3 as a potential therapeutic target against inflammation-related olfactory sensory neuronal loss and functional deficit

Jinxia Liu et al.,

Correspondence: Yiqun Yu, yu_yiqun@fudan.edu.cn

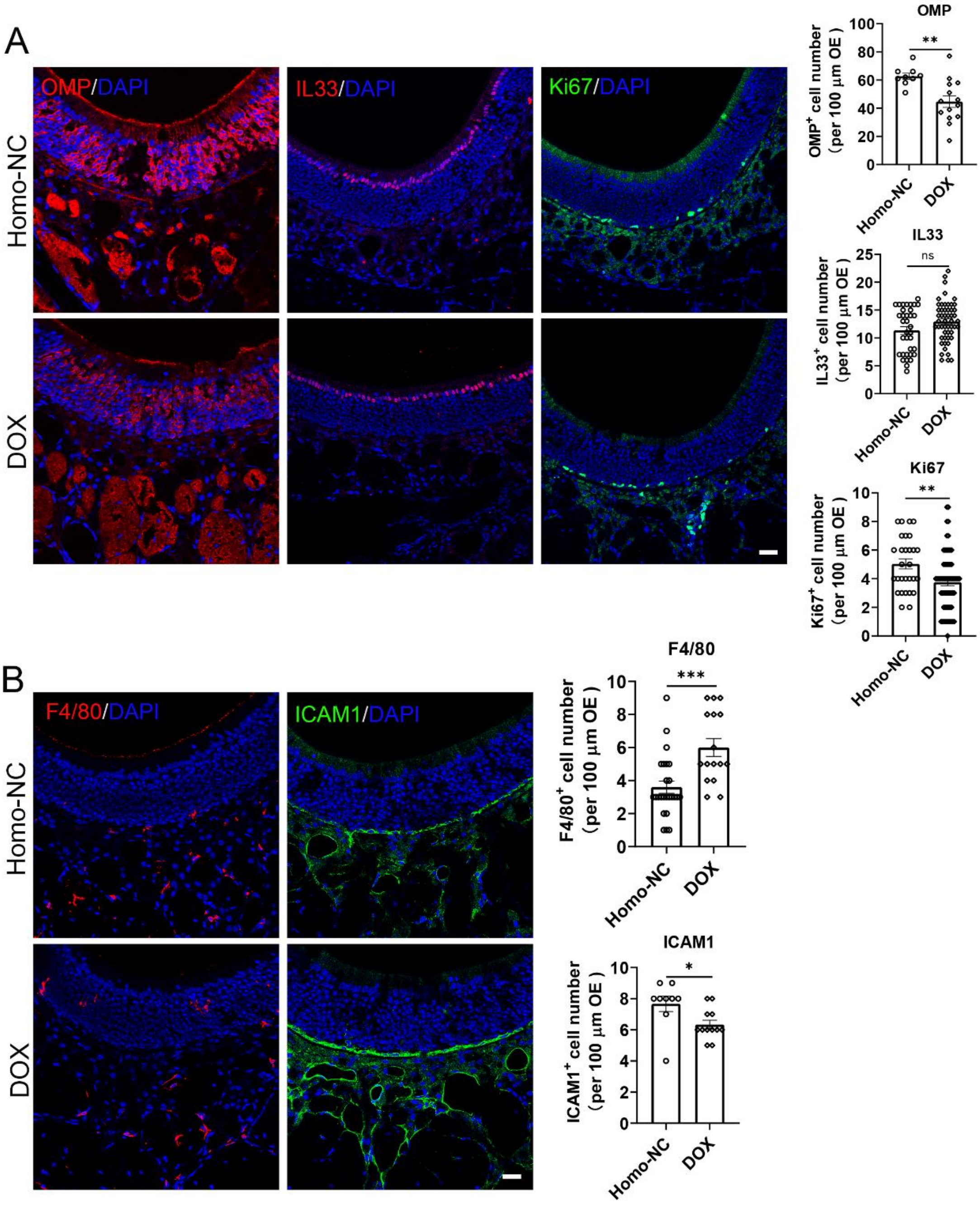


Figure S1. Cellular changes in the OE of Dox-induced olfactory inflammation mice. Confocal images and quantifications of $OMP^+$, $IL33^+$, $Ki67^+$ (A), $F4/80^+$, $ICAM1^+$ (B) cells in the OE of inducible olfactory inflammation mice with or without DOX treatment. $OMP^+$: n= 9 and 14 OE regions in control and DOX group, $IL33^+$: n= 35 and 52, $Ki67^+$: n= 29 and 69, $F4/80^+$: n= 25 and 16, $ICAM1^+$: n= 9 and 12. The statistical significances were determined by unpaired t test. ns, not significant, *$p < 0.05$, **$p < 0.01$, ***$p < 0.001$. Scales bars: 20 μm.

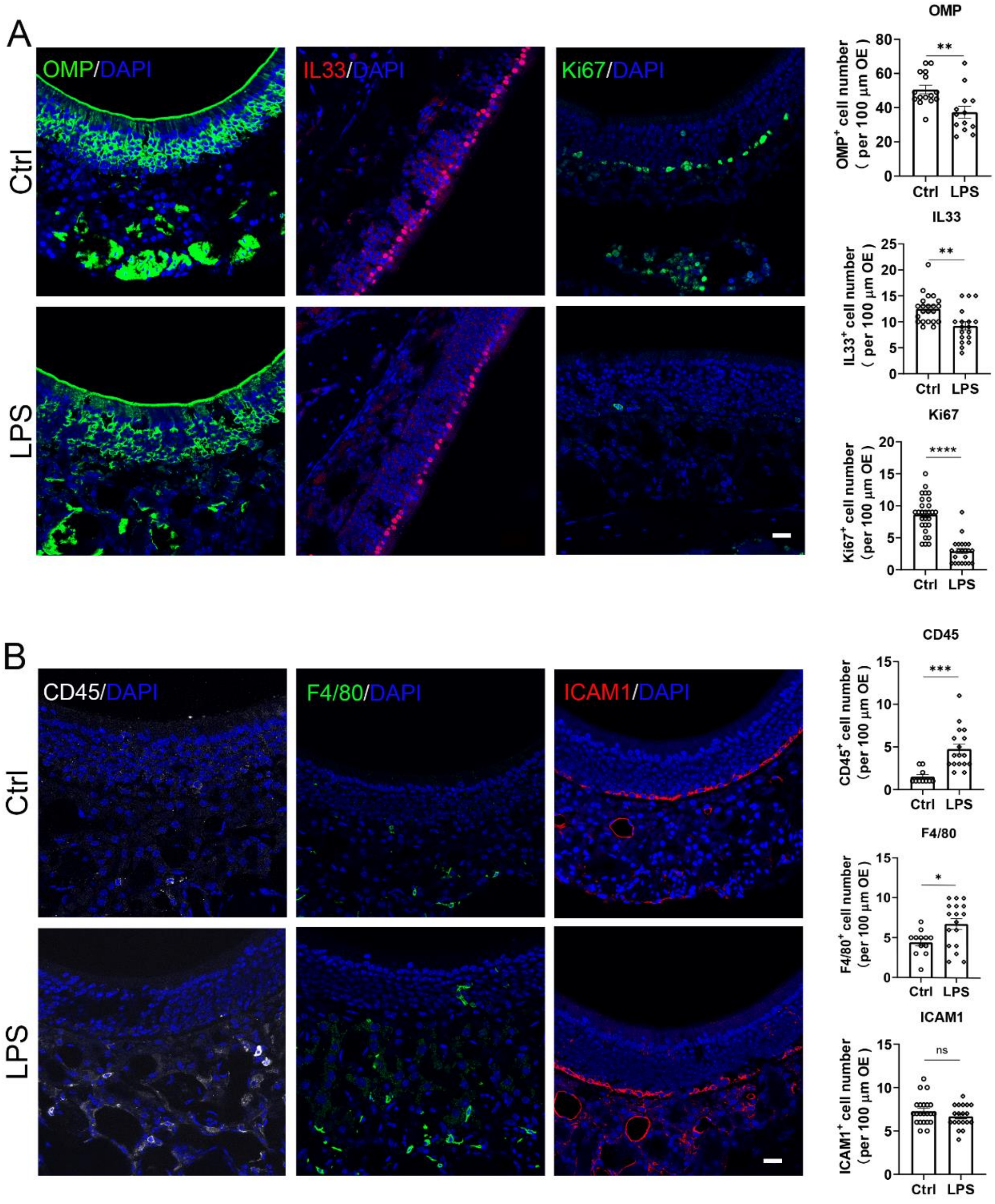


Figure S2. LPS instillation leads to cellular changes in the OE. Confocal images and quantifications of $OMP^+$, $IL33^+$, $Ki67^+$ (A), $CD45^+$, $F4/80^+$, $ICAM1^+$ (B) cells in the OE of saline and LPS-instilled mice. $OMP^+$: n= 15 and 13 OE regions, $IL33^+$: n=22 and 18, $Ki67^+$: n=28 and 31, $CD45^+$: n=10 and 17, $F4/80^+$: n=12 and 17, $ICAM1^+$: n=22 and 19. The statistical significances were determined by unpaired t test. ns, not significant, *$p < 0.05$, **$p < 0.01$, ***$p < 0.001$, **** $p < 0.0001$. Scales bars: 20 μm.

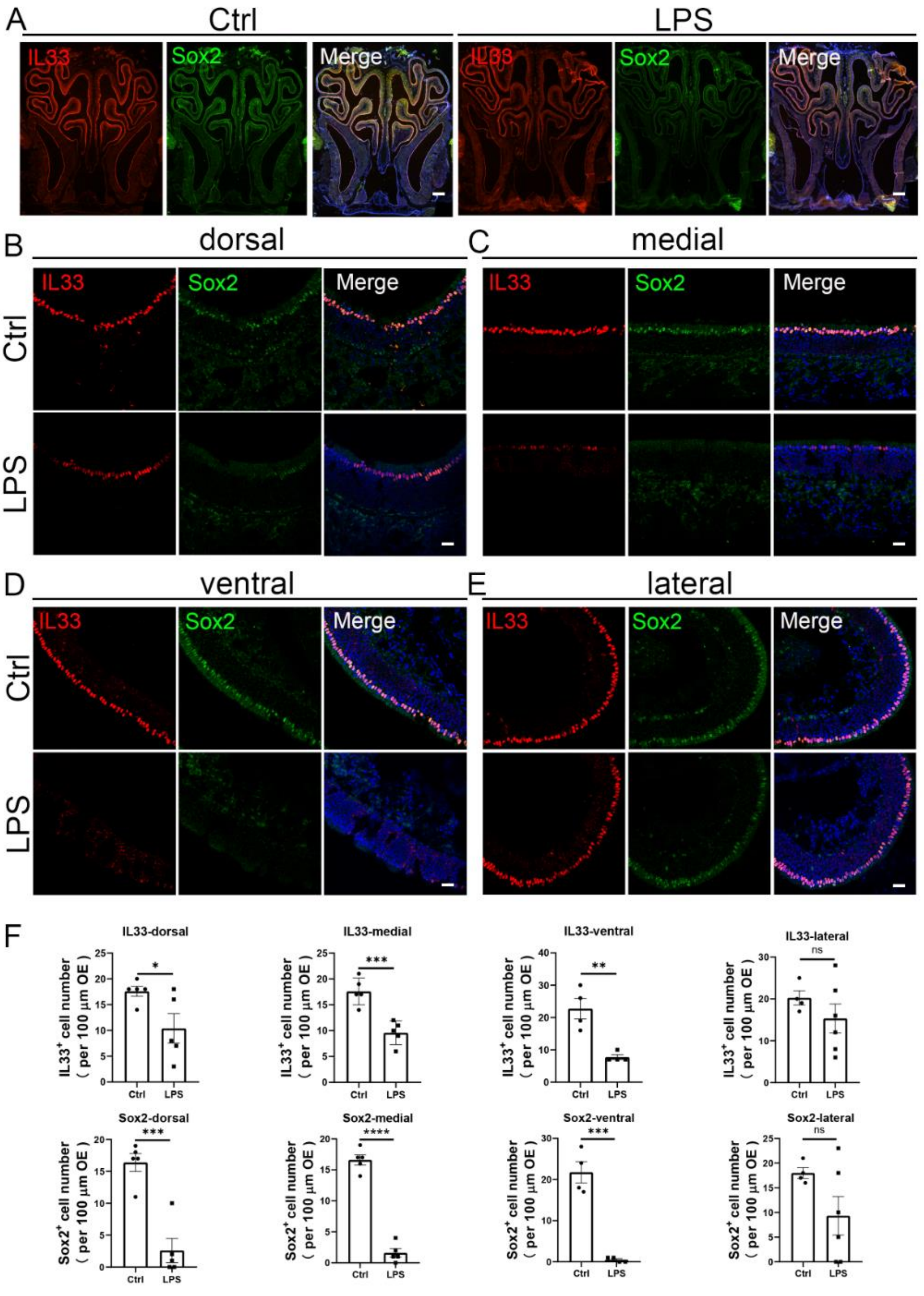


Figure S3. LPS instillation leads to regional loss of sustentacular cells in the OE. (A) Confocal images of IL33$^{+}$ and Sox2$^{+}$ cells in the whole OE section of control and LPS groups. (B-E) Confocal images of IL33$^{+}$ and Sox2$^{+}$ cells in dorsal (B), medial (C), ventral (D), and lateral (E) OE of control and LPS groups. (F) Quantification of IL33$^{+}$ and Sox2$^{+}$ cells in dorsal (B), medial (C), ventral (D), and lateral (E) OE of control and LPS groups. n = 4 – 6 regions. The statistical significances were determined by unpaired t test. ns, not significant, *$p < 0.05$, **$p < 0.01$, ***$p < 0.001$, **** $p < 0.0001$. Scales bars: 500 μm in (A), 20 μm in (B-E).

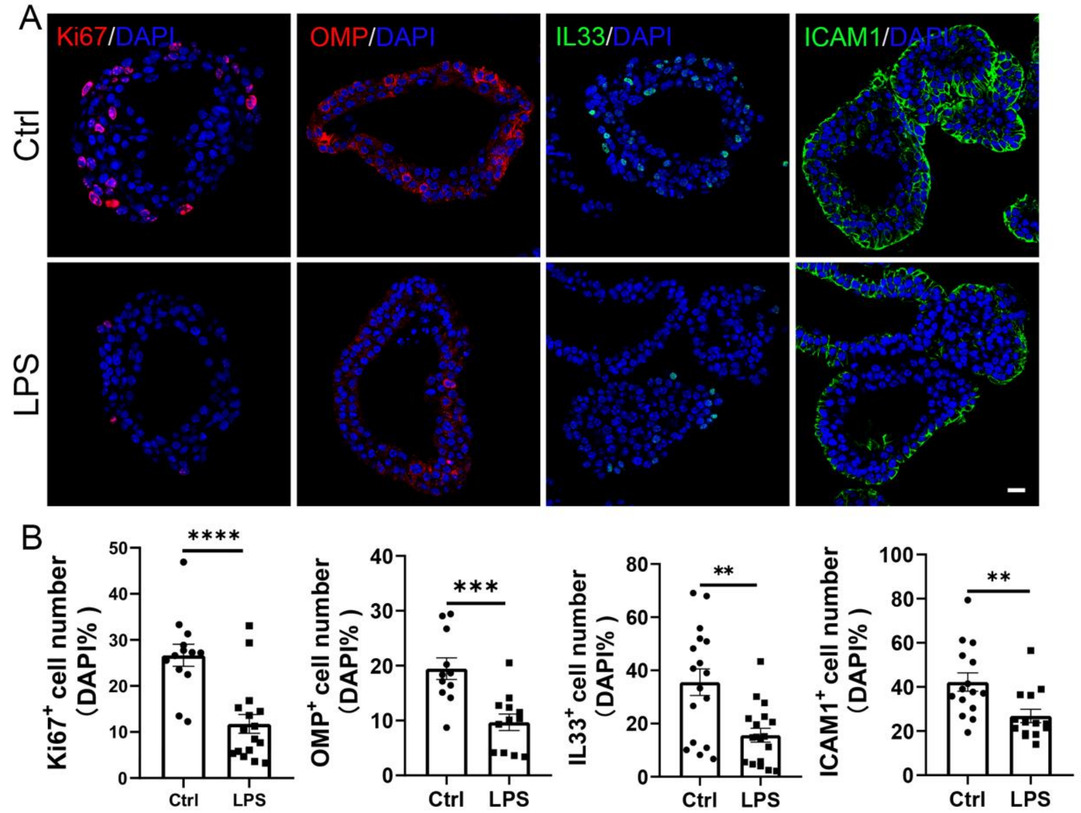


Figure S4. LPS treatment in vitro impairs cell proliferation and differentiation in OE organoids. (A, B) Confocal images (A) and quantification (B) of $Ki67^+$, $OMP^+$, $IL33^+$, and $ICAM1^+$ cells in saline or LPS-treated organoids. $Ki67^+$: n = 13 and 17 organoids in control and LPS group, $OMP^+$: n = 11 and 12 organoids, $IL33^+$: n = 17 and 18 organoids, $ICAM1^+$: n = 15 and 14 organoids. The statistical significances were determined by unpaired t test. ** $p < 0.01$, *** $p < 0.001$, **** $p < 0.0001$. Scales bar: 20 μm.

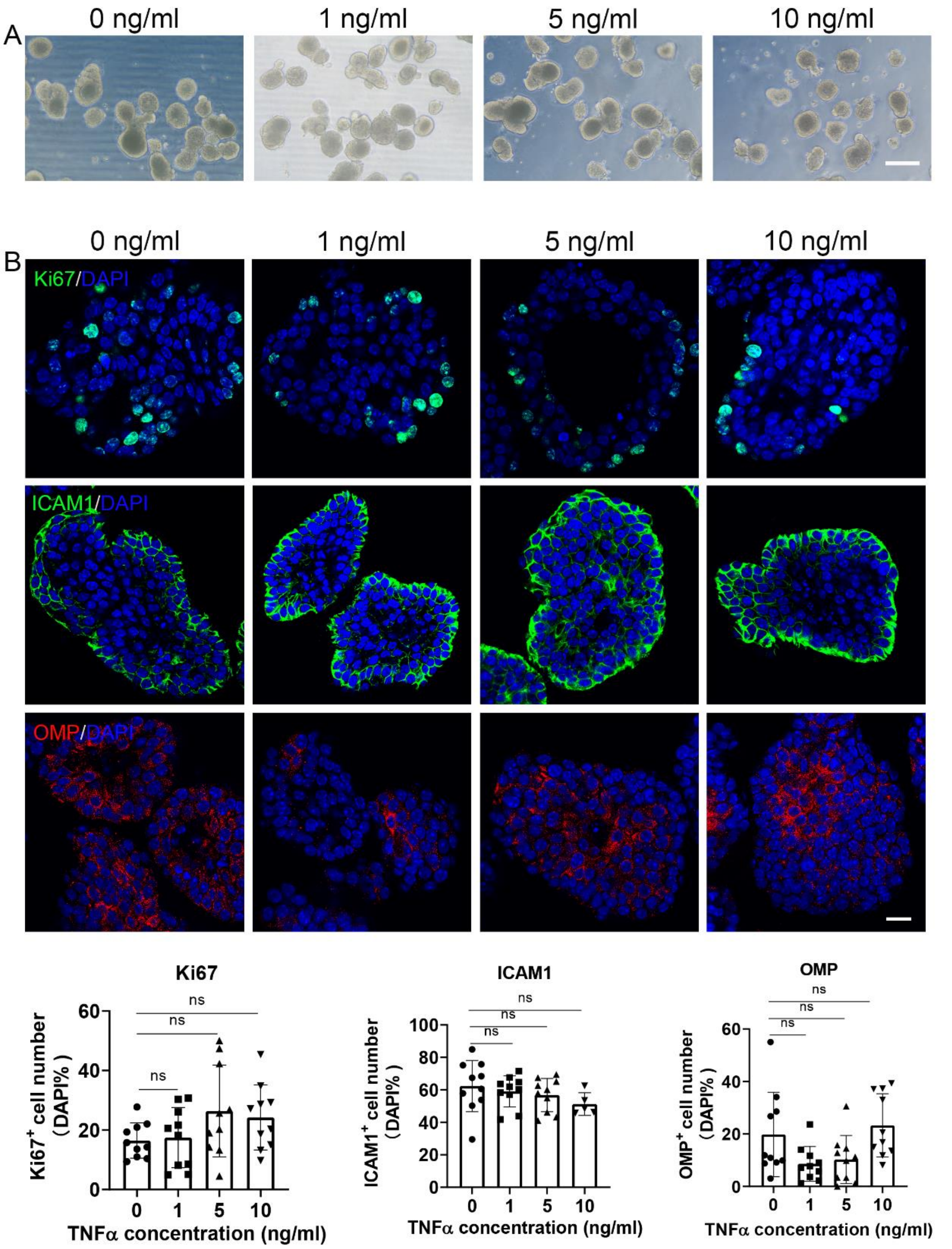


Figure S5. TNFα exposure for 24 h does not affect cell proliferation and neuronal maturation in OE organoids. (A) Microscopic images of OE organoids treated with 0, 1, 5, 10 ng/ml TNFα for 24 h. (B) Confocal images and quantification of $Ki67^+$, $ICAM1^+$, $OMP^+$ cells in OE organoids treated with 0, 1, 5, 10 ng/ml TNFα for 24 h. $Ki67^+$: n= 10 organoids in each group, $ICAM1^+$: n=10, 10, 10, 5 organoids, $OMP^+$: n= 10 organoids in each group. The statistical significances were determined by one-way ANOVA with Dunnett's multiple comparisons test. ns, not significant. Scales bars: 200 μm in (A), 20 μm in (B).

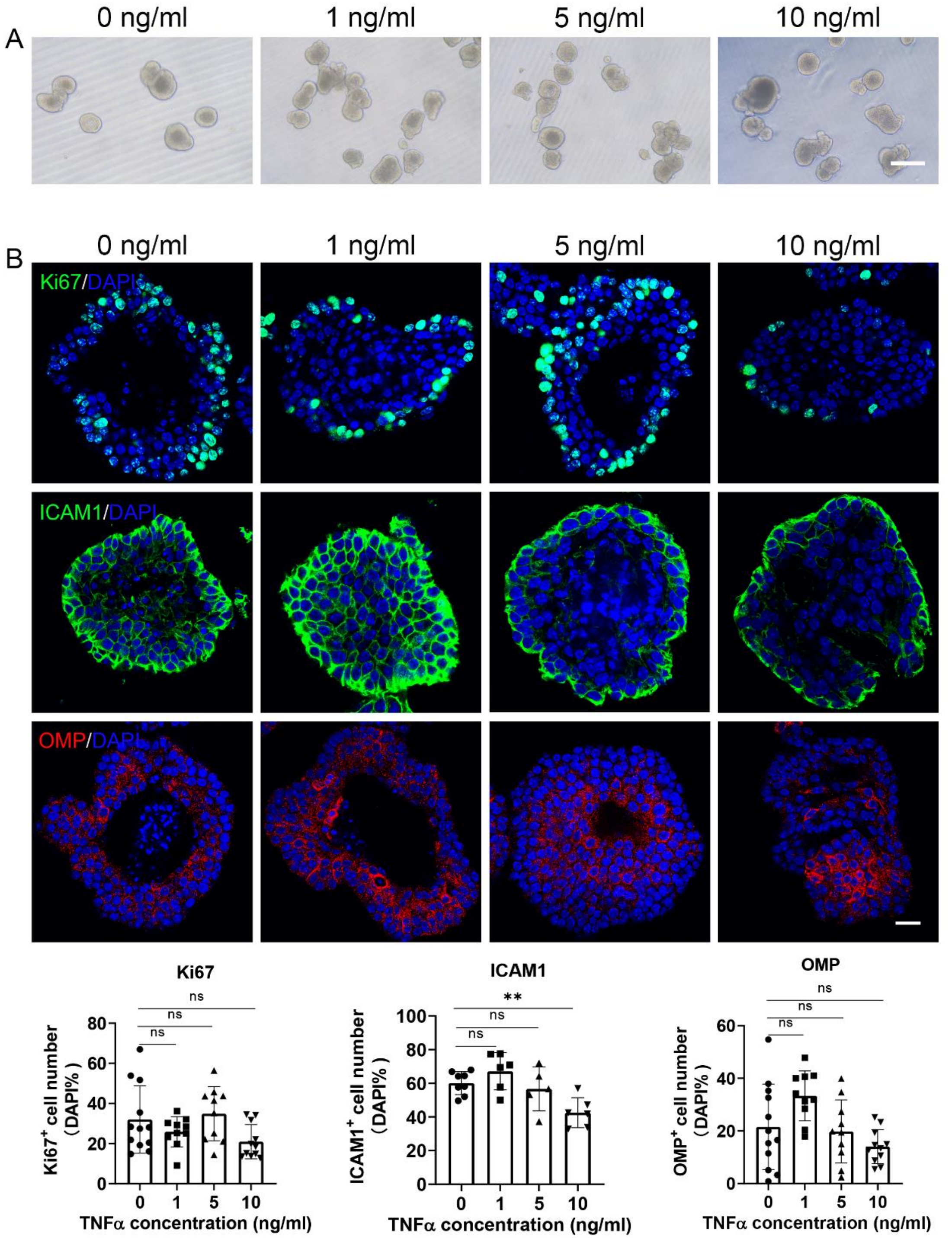


Figure S6. TNFα exposure for 48 h does not affect cell proliferation and neuronal maturation in OE organoids. (A) Microscopic images of OE organoids treated with 0, 1, 5, 10 ng/ml TNFα for 48 h. (B) Confocal images and quantification of $Ki67^+$, $ICAM1^+$, $OMP^+$ cells in OE organoids treated with 0, 1, 5, 10 ng/ml TNFα for 48 h. $Ki67^+$: n= 12, 10, 10, 11 organoids, $ICAM1^+$: n=8, 6, 5, 6 organoids, $OMP^+$: n= 12, 10, 10, 11 organoids. The statistical significances were determined by one-way ANOVA with Dunnett's multiple comparisons test. ns, not significant, ** $p < 0.01$. Scales bars: 200 μm in (A), 20 μm in (B).

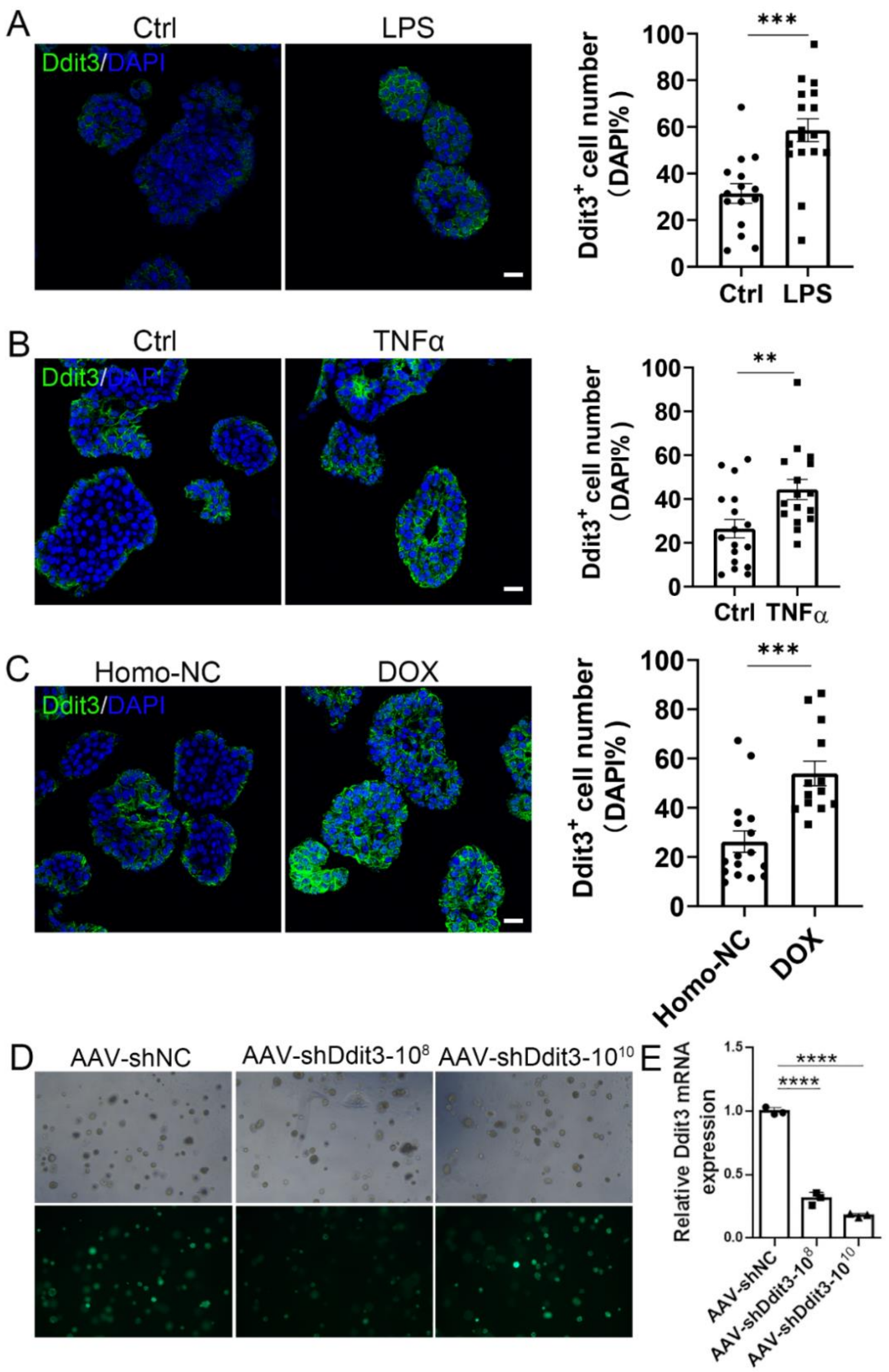


Figure S7. Increase in Ddit3$^{+}$ cells in inflammatory OE organoid models. (A-C) Confocal images and quantifications of Ddit3$^{+}$ cells in LPS- (A), TNFα- (B) treated OE organoids from WT mice, DOX-treated (C) organoids from Cyp2g1-rtTA/ TRE-TNFα mice. LPS: n= 15 and 17 organoids in control and treated group, TNFα: n= 17 and 16, DOX: n=16 and 13. (D) Microscopic images of OE organoids infected with AAV-shNC and AAV-shDdit3. (E) Quantitative PCR analysis showing Ddit3 expression levels in OE organoids infected with AAV-shNC and AAV-shDdit3. n= 3 preparations. The statistical significances were determined by unpaired t test in (A-C), and by one-way ANOVA with Dunnett's multiple comparisons test in (E). $^{**}p < 0.01$, $^{***}p < 0.001$, $^{****}p < 0.0001$. Scales bars: 20 μm.

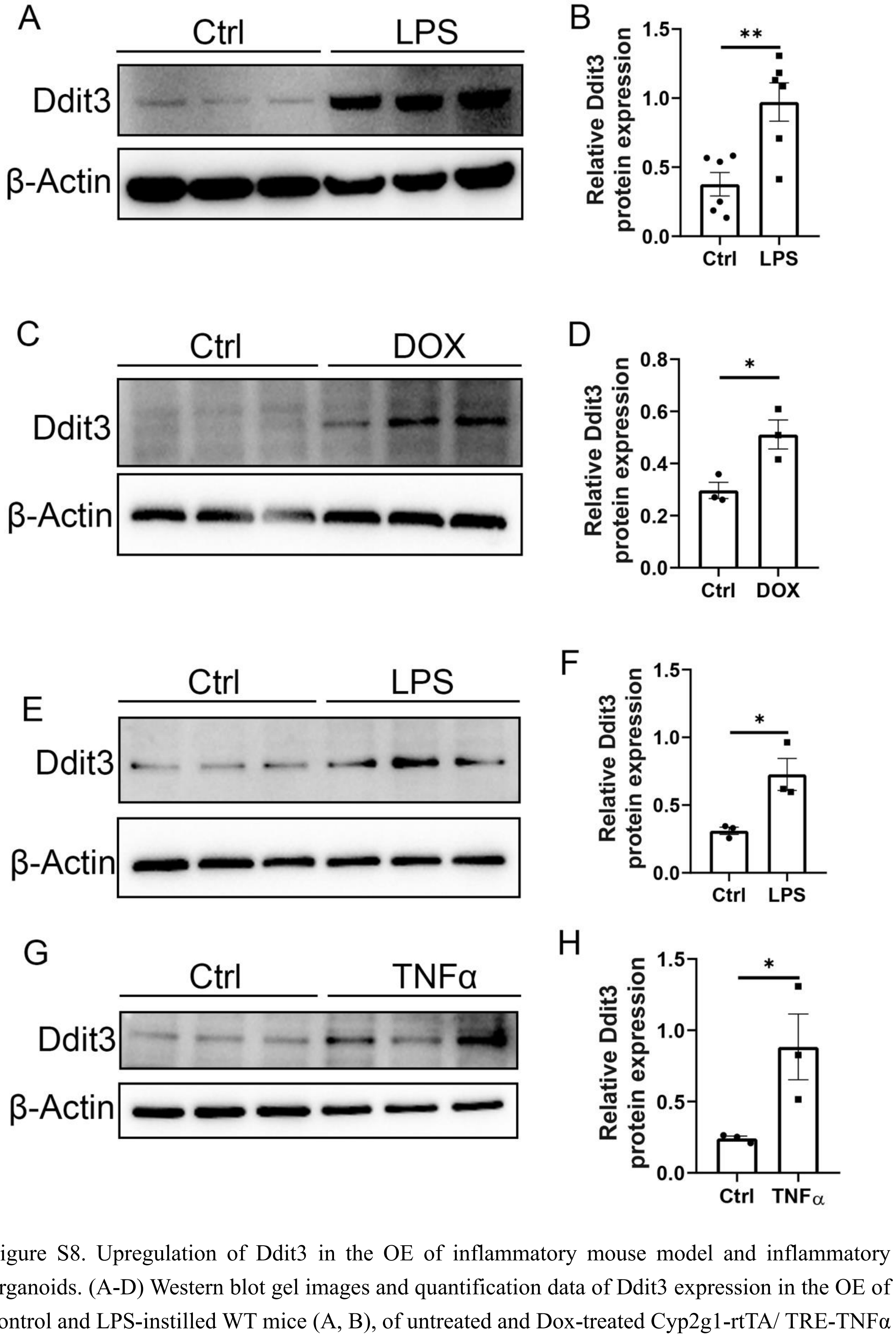


Figure S8. Upregulation of Ddit3 in the OE of inflammatory mouse model and inflammatory organoids. (A-D) Western blot gel images and quantification data of Ddit3 expression in the OE of control and LPS-instilled WT mice (A, B), of untreated and Dox-treated Cyp2g1-rtTA/ TRE-TNFα mice (C, D). (E-H) Western blot gel images and quantification data of Ddit3 expression in control and LPS-treated organoids (E, F), in untreated and TNFα-treated organoids (G, H). The statistical significances were determined by unpaired t test. *$p < 0.05$, **$p < 0.01$. n= 3 experiments.

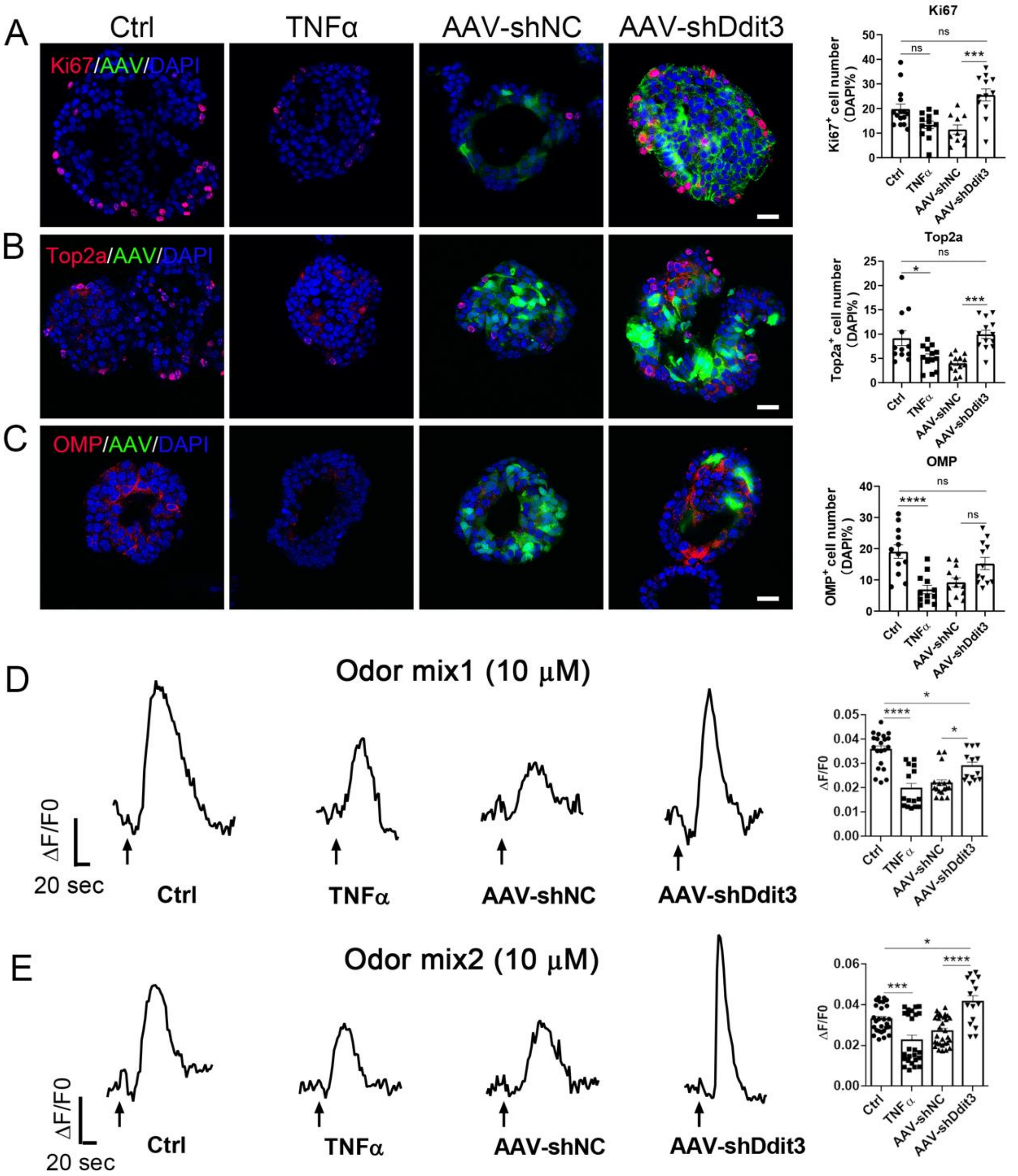


Figure S9. Ddit3 downregulation counteracts the effect of TNFα in OE organoids. (A-C) Confocal images and quantification of Ki67+ (A), Top2a+ (B), OMP+ (C) cells in OE organoids treated with saline or TNFα, and in TNFα-treated organoids infected with AAV-shNC or AAV-shDdit3. Ki67$^+$: n= 15, 12, 10, 12 organoids in control, LPS, LPS+shNC, LPS+shDdit3 group, Top2a$^+$: n= 12, 13, 13, 13 organoids, OMP$^+$: n= 12 organoids in each group. (D, E) Representative calcium imaging curves of OE organoids treated with saline or TNFα, and in TNFα-treated organoids infected with AAV-shNC or AAV-shDdit3, stimulated with odor mix1 (D) and mix2 (E). Quantifications of calcium imaging data were shown at the right. Mix1: n= 21, 15, 17, 13 recordings in control, TNFα, TNFα+shNC, TNFα+shDdit3 group, Mix2: n= 27, 25, 30, 15 recordings. The statistical significances were determined by one-way ANOVA with Tukey's multiple comparisons test. ns, not significant, *p < 0.05, ***p < 0.001, **** p < 0.0001. Scales bars: 20 μm.

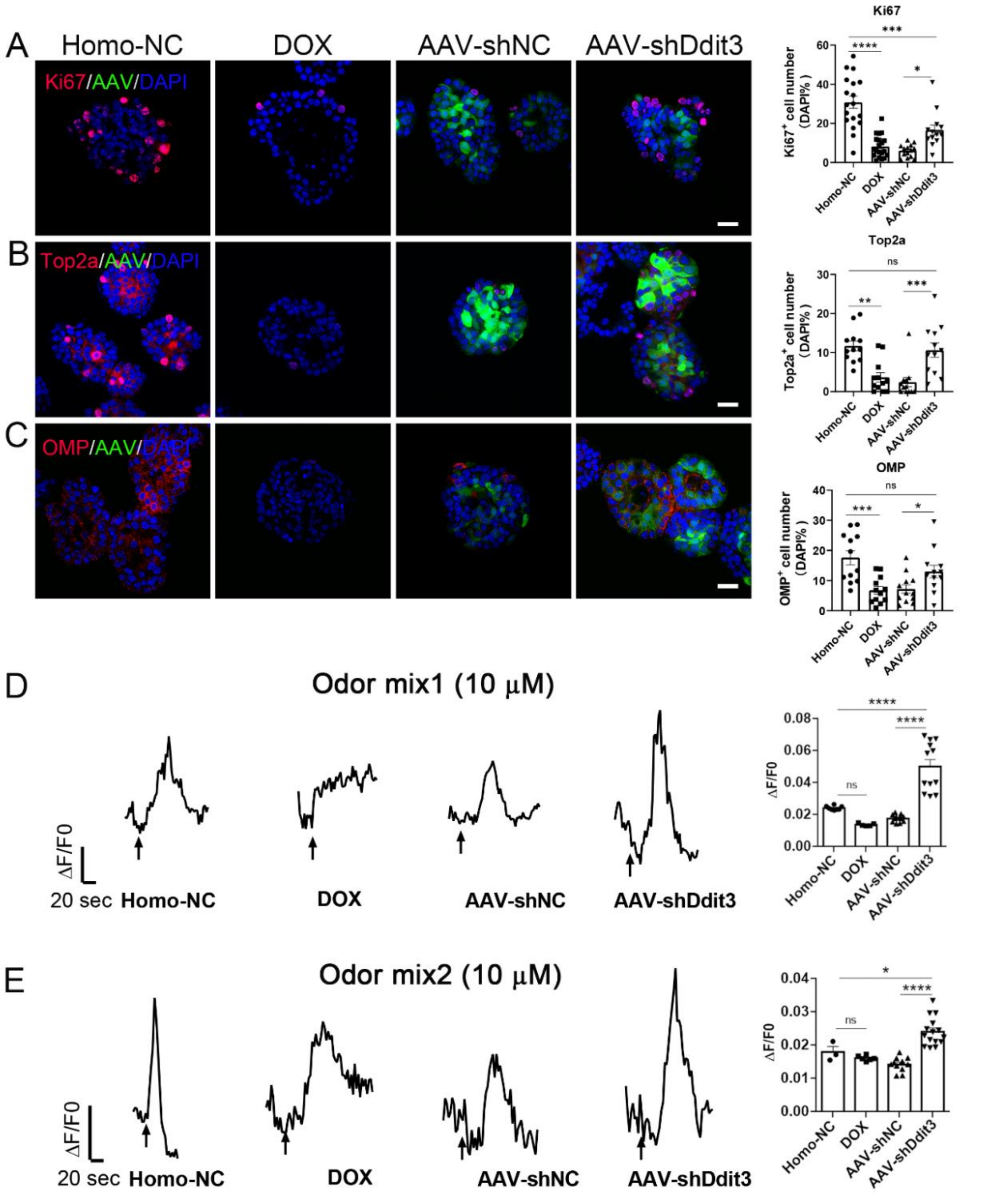


Figure S10. Ddit3 downregulation counteracts the effect of inducible TNFα in OE organoids. (A-C) Confocal images and quantification of Ki67+ (A), Top2a+ (B), OMP+ (C) cells in OE organoids treated with saline or DOX, and in DOX-treated organoids infected with AAV-shNC or AAV-shDdit3. $Ki67^{+}$: n= 18, 20, 14, 13 organoids in control, LPS, LPS+shNC, LPS+shDdit3 group, $Top2a^{+}$: n= 12 organoids in each group, $OMP^{+}$: n= 12, 12, 12, 11 organoids. (D, E) Representative calcium imaging curves of OE organoids treated with saline or DOX, and in DOX-treated organoids infected with AAV-shNC or AAV-shDdit3, stimulated with odor mix1 (D) and mix2 (E). Quantifications of calcium imaging data were shown at the right. Mix1: n= 8, 5, 12, 12 recordings in control, DOX, DOX+shNC, DOX+shDdit3 group, Mix2: n= 3, 6, 11, 15 recordings. The statistical significances were determined by one-way ANOVA with Tukey's multiple comparisons test. ns, not significant, *p < 0.05, **p < 0.01, ***p < 0.001, **** p < 0.0001. Scales bars: 20 μm.

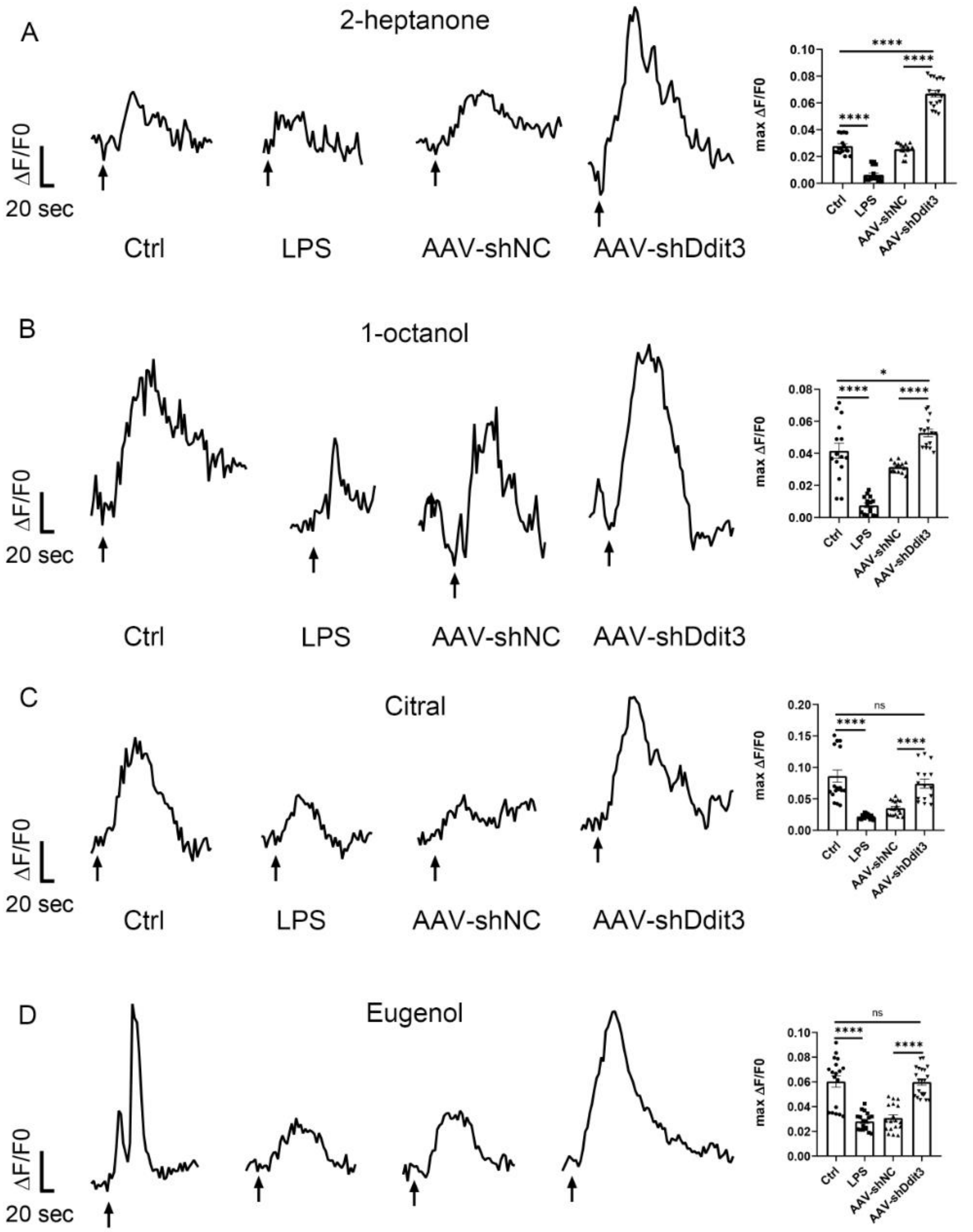


Figure S11. Ddit3 downregulation recovers response to single odorant in LPS-treated organoids. (A-D) Representative calcium imaging curves of OE organoids treated with saline or LPS, and in LPS-treated organoids infected with AAV-shNC or AAV-shDdit3, stimulated with 2-heptanone (A), 1-octanol (B), citral (C), eugenol (D). Quantifications of calcium imaging data were shown at the right. 2-heptanone: n= 16, 16, 14, 18 recordings in control, LPS, LPS+shNC, LPS+shDdit3 group, 1-octanol: n= 15 recordings in each group, citral: n= 18, 18, 17, 15 recordings, eugenol: n= 18, 18, 18, 21 recordings. The statistical significances were determined by one-way ANOVA with Tukey's multiple comparisons test. ns, not significant, *p < 0.05, *** p < 0.0001.

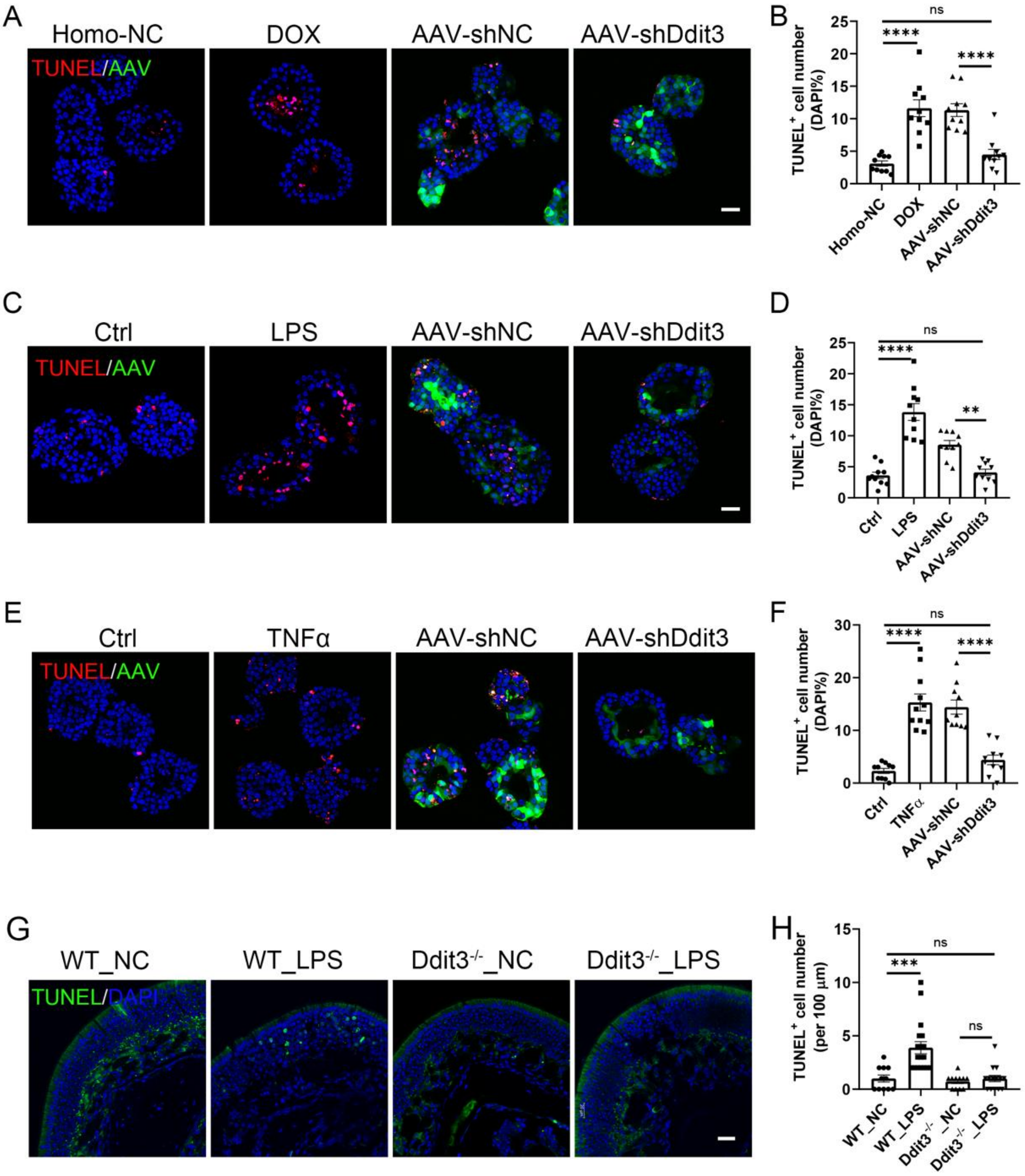


Figure S12. Ddit3 deficiency or downregulation alleviates the apoptosis in the inflammatory models. (A-F) Confocal images and quantification of $TUNEL^+$ cells in Dox-induced (A, B), LPS-treated (C, D), TNFα-treated (E, F) organoids with AAV-NC or AAV-shDdit3 infection. n= 10 organoids in each group. (G, H) Confocal images (G) and quantification (H) of $TUNEL^+$ cells in the OE of saline or LPS-instilled WT and $Ddit3^{-/-}$ mice. n= 11, 18, 11, 14 OE regions in WT_NC, WT_LPS, $Ddit3^{-/-}$_NC, $Ddit3^{-/-}$_LPS group. The statistical significances were determined by one-way ANOVA with Tukey's multiple comparisons test. ns, not significant, **$p < 0.01$, ***$p < 0.001$, **** $p < 0.0001$. Scales bars: 20 μm.

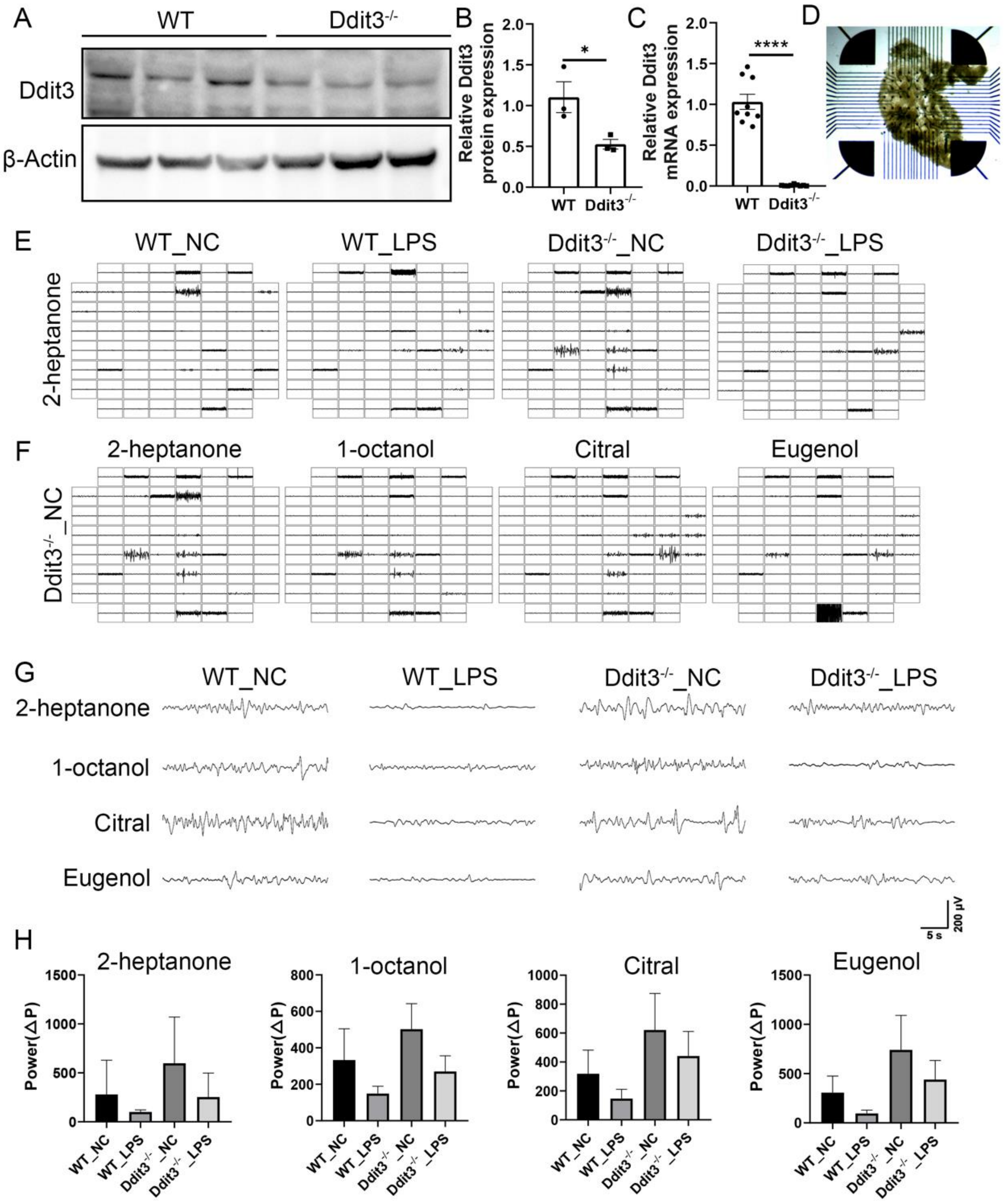


Figure S13. Ddit3 deficiency recovers the electrophysiological response to single odorants in the OE of LPS-instilled mice. (A, B) Western blot band image (A) and quantification (B) showing Ddit3 expression level in the OE of WT and Ddit3$^{-/-}$ mice. (C) Quantitative PCR data of Ddit3-mRNA expression level in the OE of WT and Ddit3$^{-/-}$ mice. (D) Images of MEA system with OE tissue. (E) Original response curves to 2-heptanone in multiple OE regions of saline or LPS-instilled WT mice, and saline or LPS-instilled Ddit3$^{-/-}$ mice. (F) Original response curves to four different single odorants in the OE of saline-treated Ddit3$^{-/-}$ mice. (G) Representative response curves to four single odorants by MEA in the OE of saline or LPS-instilled WT mice, and saline or LPS-instilled Ddit3$^{-/-}$ mice. (H) Quantification of the ΔP of the response to 2-hepatanone, 1-octanol, citral, eugenol by MEA. n= 4 OE tissues in each group.
